# The utilization of ions in seawater for electrocatalysis

**DOI:** 10.1093/nsr/nwaf461

**Published:** 2025-10-29

**Authors:** Linsen Huang, Deyu Bao, Yunling Jiang, Klaus Regenauer-Lieb, Yao Zheng, Shi-Zhang Qiao

**Affiliations:** School of Chemical Engineering, The University of Adelaide, Adelaide, SA 5005, Australia; School of Chemical Engineering, The University of Adelaide, Adelaide, SA 5005, Australia; School of Chemical Engineering, The University of Adelaide, Adelaide, SA 5005, Australia; WA School of Mines: Minerals, Energy and Chemical Engineering, Curtin University, Perth, WA 6151, Australia; School of Chemical Engineering, The University of Adelaide, Adelaide, SA 5005, Australia; School of Chemical Engineering, The University of Adelaide, Adelaide, SA 5005, Australia

**Keywords:** ions, seawater, electrocatalysis, reactions, energy conversion

## Abstract

Electrocatalysis in water offers a sustainable pathway for synthesizing hydrogen and hydrocarbons, leveraging water as a source of protons and hydroxides. However, the inherent presence of ions in water significantly influences the adsorption of active species, often disrupting electrocatalytic performance. While strategies have advanced to repel interfering ions and mitigate their adverse effects in seawater electrocatalysis, recent findings reveal that certain seawater ions can enhance electrocatalytic processes. This enhancement occurs either by promoting the adsorption of crucial reaction intermediates or by directly participating as reactants. Despite this potential, the underlying mechanisms and practical applications of these ion-involved reactions remain poorly understood and lack systematic evaluation. This review provides a timely appraisal of electrochemical reactions that strategically utilize seawater ions. It highlights recent advancements in methodologies and strategies within this emerging field. Firstly, we delve into the designs and mechanisms enabling chloride utilization in various electrocatalytic reactions. Next, we discuss intelligent protocols for sodium utilization, including asymmetric designs, aqueous alternating and cascade seawater electrocatalysis. Subsequently, we elucidate the critical role of local pH limitations and the impact of external forces on catalysts for direct seawater electrocatalysis. Finally, the electrochemical extraction of valuable resources such as uranium and lithium is summarized, with a focus on innovative electrode modifications and optimized cell configurations.

## Teaser

This review highlights recent advancements and future opportunities on utilizing seawater ions to enhance selectivity, activity, and stability across diverse electrocatalytic reactions.

## INTRODUCTION

Among the synthesis technologies of various commodity chemicals such as hydrogen, hydrocarbons or oxygenates, electrocatalysis in aqueous solutions is gaining prominence due to its cost-effectiveness and potential for net-zero emissions [1–3]. It is acknowledged that water as the source of protons and hydroxides serves the electrocatalysis process. To stabilize the pH and enhance the conductivity of bulk aqueous electrolyte, ionic compounds or additives are typically introduced into water. These free ions in water exert direct or indirect detrimental effects on electrochemical reaction performance by manipulating the proton and hydroxide supply. For example, certain ions (e.g. Mg^2+^, Cl⁻) in seawater-based electrocatalysis can poison active sites of catalysts, thereby diminishing electrocatalytic activity and longevity. Therefore, the complex synergy among multiple ions in seawater, including a wide range of soluble inorganic ions (Table 1), along with microorganisms, small organic molecules and dissolved gases [4,5], presents considerable challenges for electrocatalysis.

**Table 1.** Ion composition and concentration of seawater.

Ions	Concentration (mol L^−1^)	Ions	Concentration (mol L^−1^)
Cl^−^	∼0.55	Sr^2+^	∼0.000 09
SO_4_^2−^	∼0.028	UO_2_^2+^	∼1 × 10⁻⁸
Br^−^	∼0.00084	Cu^2+^	∼10⁻⁸
F^−^	∼0.00007	Pb^2+^	∼10⁻¹²
Na^+^	∼0.47	B(OH)_3_	∼0.00032
Mg^2+^	∼0.053	B(OH)_4_^−^	∼0.00010
Ca^2+^	∼0.010	HCO_3_^−^	∼0.002
K^+^	∼0.010	CO_3_^2−^	∼0.0002 to 0.0003
Li^+^	∼0.0025	OH^−^	∼10⁻^7^ to 10⁻^6^

To tackle those challenges, significant advances in both electrochemical systems and catalysts design have thus developed to mitigate the negative impacts of ions; a fairly universal strategy is the surface repulsion of ions through surface modifications [6,7]. However, little direct evidence has uncovered the surface repulsion due to the occurrence of surface charges on the electrodes during the reaction. More precisely, the traditional ion-repelling strategies can be deemed as a reasonable hypothesis rather than a confirmed mechanism. In sharp contrast, leveraging specific ions in seawater electrocatalysis can enhance catalyst performance, selectivity and value-added by-product formation, rather than merely mitigating ion interference. With the rapid development of *in situ* techniques, the latest advancement of electrocatalysis based on seawater has exploited the utilization of ions (e.g. adsorption of ions) to counter its detrimental effects. For example, the interaction between an Ir/CoFe-layered double hydroxide (LDH) catalyst surface and Cl⁻ in seawater not only facilitates the oxygen evolution reaction (OER) activity by lowering the activation energy, but also boosts the stability of catalysts for alkaline seawater electrocatalysis systems [8]. Despite these advancements, harnessing seawater ions for electrocatalysis requires the development of highly selective and stable catalysts, as well as sophisticated electrolyser systems capable of withstanding the harsh and variable conditions present in natural seawater [9,10].

The primary challenges for electrocatalysis in natural seawater are alkali-metal ion (Mg^2+^/Ca^2+^)-derived insoluble precipitates at the cathode [5,11], along with chloride-induced corrosion at the anode [12,13]. A promising approach to mitigate these problems involves turning the detrimental ions into useful resources—e.g. recycling high-value metal ions such as Mg²⁺ during the electrocatalytic process or converting Cl⁻ into active chlorine disinfectants in direct seawater electrolysis [14,15]. However, these methodologies introduce new complexities for the mechanistic understanding of these ion-involved electrochemical reactions when using natural seawater as a feedstock. To date, a comprehensive summary of these reactions remains lacking.

**Figure 1.**
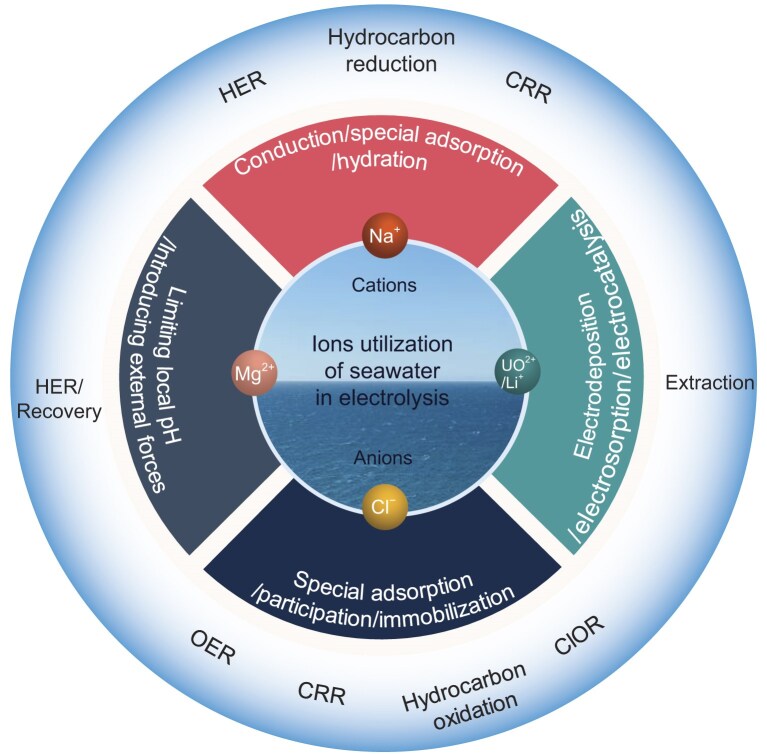
Scheme of utilization of seawater ions in various electrocatalysis processes.

In contrast to earlier efforts in seawater electrocatalysis, which primarily focused on removing or repelling interfering ions through additives or catalyst optimization, we highlight recent advancements that actively harness or recycle these ions to enhance the performance of seawater electrocatalysis. These innovations effectively mitigate the detrimental effects of inorganic salts while unlocking functional benefits. In this review, we discuss key issues and pertinent research concerning the utilization of ions in seawater electrocatalysis and the advanced electrochemical catalysts and electrolyser designs (Fig. 1). First, we explore the roles of Cl^−^, which are determined as specific adsorption on the catalyst surface, participation in the electrochemical reaction and immobilization in catalysts across various electrochemical processes. Second, we elaborate on sodium-ion (Na^+^) utilization patterns of electrochemical reactions via conduction, special adsorption and hydration effects. Third, tuning the local pH and introducing external forces over electrocatalysts are discussed to achieve the co-production of hydrogen and magnesium hydroxide in direct seawater electrocatalysis. Finally, we provide a brief description of the electrochemical extraction of uranium from seawater by using an electrodeposition, electrosorption and electrocatalysis process, as well as lithium recovery enabled by using specialized cell configurations.

## RATIONAL UTILIZATION OF ANIONS

The concentration of anions in seawater is ranked as follows: Cl^−^ > SO_4_^2−^ > HCO_3_^−^ > Br^−^ [5,10]. Among them, oxyanion additives (SO_4_^2−^ or HCO_3_^−^) in alkaline electrolytes have been shown to improve anodic catalyst stability and even repel Cl^−^, thereby offering protection against chloride-induced corrosion [16,17]. However, the challenge posed by the chemical attack of Cl^−^ on catalyst active sites remains a significant hurdle in seawater electrocatalysis. At present, most studies have focused on creating anticorrosion layers to weaken or mitigate the inimical effects of Cl⁻ on catalysts [7,18–20]. Nevertheless, beyond protective measures, more intriguing strategies are emerging that leverage the unique properties of Cl^−^ to enhance the stability of the anodic catalyst during the OER or to serve as a promoter for other electrochemical reactions. In this section, we classify five distinctive aspects of Cl^−^ utilization that have been recognized as key breakthroughs in advancing seawater electrocatalysis. These include the effective use of Cl^−^ to enhance the OER performance in an alkaline seawater system, direct conversion of chloride into chlorine during seawater electrocatalysis, light hydrocarbon electrooxidation in the presence of chloride, specific adsorption of Cl^−^ for carbon-dioxide-reduction reaction and other Cl^−^-involved reactions.

### Effective utilization of Cl^−^ for OER in alkaline seawater electrocatalysis

The presence of Cl⁻ in seawater continues to pose a significant challenge during practical electrocatalysis operations, as the chloride oxidation reaction (ClOR) competes with the OER and the chemical coordination interaction between the Cl^−^ and the catalysts can degrade the catalyst and further corrode the electrode [9,21]. To maximize selective OERs over ClORs, the alkali ($800 tonnes^−1^ KOH) additive is recommended to lower the onset potential of OERs and mitigate the effects of Mg^2+^ and Ca^2+^ in seawater. However, achieving an efficient OER in alkaline seawater electrocatalysis remains challenging, as ClORs can readily outcompete OERs at industrial current densities. Most investigations concern establishing an *in situ*-derived anion-rich surface, creating a solid protection layer or incorporating ions into the catalyst layers of catalysts to repel Cl^−^ effectively [6,7,17,18,22–25]. However, limited studies have been aimed at utilizing the natural presence of Cl^−^ in seawater to promote the activity and durability of anodic electrocatalysts.

**Figure 2.**
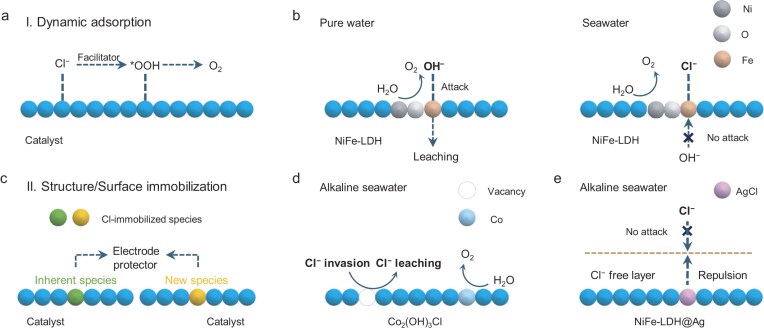
Cl^−^ utilization scenario in alkaline seawater electrocatalysis. (a, c) Schematic configurations of (a) dynamic adsorption and (c) structure or surface immobilization of Cl^−^. (b) OER mechanism evolution via Cl^−^ adsorption over NiFe-LDH [28]. (d, e) Strategies of (d) structure immobilization of Cl^−^ over Co_2_(OH)_3_Cl [29] and (e) surface immobilization of Cl^−^ over NiFe-LDH@Ag [30].

The direct utilization of Cl⁻ in seawater electrocatalysis stems from its dynamic adsorption on the active sites of electrocatalysts during water dissociation. This process enhances the adsorption of surface-bound OOH (*OOH) intermediates, thereby improving the efficiency of the OER, as illustrated in Fig. 2a [26,27]. For example, Duan *et al*. [8] tailored atomic iridium (Ir) on a CoFe-LDH catalyst to promote Cl^−^ adsorption and modulate the electronic structure of Ir. This dynamic Cl^−^ adsorption on the Ir was evidenced by using *in situ* Raman spectroscopy and theoretical analyses, revealing that Ir–OH/Cl coordination could reduce the energy barrier to form *OOH and thus improve OER performance in an alkaline electrolyte of 6 M NaOH with 2.8 M NaCl, achieving an impressive OER performance with 100% catalytic selectivity at 400–800 mA cm^−2^ over 1000 h. Similarly, Liu *et al*. [28] used a typical catalyst of NiFe-LDH that featured Cl^−^ adsorption at iron sites for alkaline seawater electrocatalysis. *In situ* differential electrochemical mass spectrometry (DEMS) confirmed that lattice oxygen in NiFe-LDH takes part directly in oxygen formation—a process known as the lattice oxygen mechanism. This mechanism is accompanied by an increase in Fe-ion leaching due to OH^−^ attack and stability loss in pure water (Fig. 2b, left). However, in alkaline seawater, *in situ*-attenuated total reflectance surface-enhanced infrared absorption spectroscopy demonstrated increased *OOH intermediate for OERs, known as the adsorbate evolution mechanism (AEM). Cl^−^ adsorption at Fe sites avoided OH^−^ attack and reduced Fe leaching (Fig. 2b, right). As a result, a commercial-scale alkaline electrolyser (120 cm^2^) using the NiFe-LDH anode achieved 100 h of stability at 200 mA cm^−2^.

Beyond specialized Cl^−^ adsorption on catalysts in seawater, functional catalysts harnessing Cl^−^ (structure/surface immobilization) can also defeat Cl^−^ permeation and corrosion (Fig. 2c). In contrast to dynamic Cl^−^ adsorption during OERs, Cl^−^ was deliberately used to immobilize into anodic materials for anticorrosion. As shown in Fig. 2d, Zhuang *et al*. [29] implemented a structural buffer engineering strategy with Co_2_(OH)_3_Cl to reach high OER selectivity and long durability in alkaline seawater. The as-described structural buffer continuously leaches lattice Cl^−^ during electrocatalysis and the resulting vacancies are filled by the Cl^−^ of seawater. This dynamic balance of Cl^−^ leaching and invasion keeps the structure and activity of the Co_2_(OH)_3_Cl, allowing it to maintain a current density of 330.5 mA cm^–2^ at a low potential of 1.63 V sustained over 60 000 s without a significant decrease at an initial current density of 130 mA cm^–2^. Furthermore, Xu *et al*. [30] developed a surface chloride immobilization (SCI) strategy to obtain remarkable long-term durability at a current density of 400 mA cm^–2^ in alkaline seawater over 2500 h for NiFe-LDH@Ag. *In operando* Raman studies uncovered that oxidized Ag nanoparticles reacted with Cl^−^ in seawater to form insoluble AgCl. This stable AgCl species was key to repelling Cl^−^, creating a Cl^−^-free layer near the anodic surface that inhibited severe Cl^−^ migration to the anode (Fig. 2e). Importantly, the proposed SCI method was also demonstrated on phosphatized NiFe-LDH (NiFeP) as the substrate.

### Direct conversion of Cl^−^ into chlorine in seawater electrocatalysis

**Figure 3.**
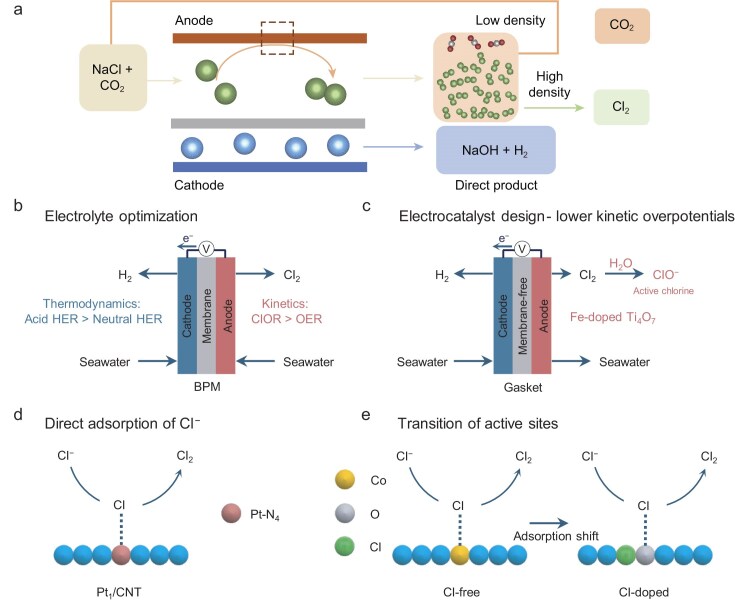
(a) Schematic of current chlor-alkali process. Reproduced with permission from [34]. Copyright 2023, Springer Nature. (b–e) Designed strategies to effectively promote ClOR in seawater, which are described as (b) electrolyte optimization and (c) electrocatalyst design, (d) direct adsorption of Cl^−^ and (e) transition of Cl^−^ active site.

The ClOR is typically believed to be a side reaction in seawater electrocatalysis because it is the companion reaction to the OER. However, Cl_2_ produced from ClORs is a value-added chemical precursor for various applications, including polymer synthesis, pharmaceutical manufacture, disinfection goods production and wastewater treatment [31,32]. Although the current chlor-alkali process is well-established technology, it relies on a highly concentrated NaCl electrolyte (≥4.0 M) to secure chloride oxidation efficiency and selectivity, which aggravates the energy input and freshwater consumption (Fig. 3a) [33,34]. Seawater, with a natural salinity of ∼35 g L^−1^ (∼0.6 M Cl^−^), serves as a widespread feedstock for ClORs and hydrogen evolution reaction (HERs). However, with such a low concentration of NaCl, this process suffers from the competing OER, which leads to low Cl_2_ selectivity, activity and stability of catalysts over the anode during the ClOR in natural seawater electrocatalysis [35]. To overcome those problems, an acid–saline hydride electrocatalysis system has been developed for the co-production of Cl_2_ and H_2_ at low cell voltages. The matched reaction with kinetics and thermodynamics at both electrodes in an acidic environment can obtain higher current densities with lower cell voltages in comparison with neutral conditions (Fig. 3b). Zhu *et al*. [36] decoupled the electrolytes by using a monolithic TiRu/Ti electrode, affording a low cell potential of 1.59 V at 10 mA cm^−2^ with an energy-consumption saving of 27.7% compared with that of a neutral electrocatalysis system (2.20 V). In addition, this system employed naturally acidified seawater as the electrolyte and bipolar membrane as the membrane, avoiding Ca/Mg-based precipitates at the cathode while allowing real-time active chlorine for sterilization and pea-sprout production at the anode.

In addition to optimizing the electrolyte, direct seawater electrocatalysis promises great potential due to its economic feasibility and operability. For instance, an Fe-doped Ti_4_O_7_ anode, using natural seawater as the electrolyte, was fabricated for chlorine disinfectant synthesis [15]. Mechanism analysis revealed that the electrophilicity of lattice oxygen in Fe-doped Ti_4_O_7_ facilitates site-selective chloride activation at lower overpotentials compared with OERs. To obviate the potentially increased cell resistance and power losses resulting from the precipitation of Mg^2+^ and Ca^2+^, a membrane-free electrolyser was designed for the applicability of this material. Consequently, this seawater-splitting device delivers an HER at the cathode and a ClOR at the anode (Fig. 3c), and further integrates with commercial silicon photovoltaic cells for an active chlorine-production rate of 3.15 mg min^−1^ for simulated ballast-water disinfection. Following the principle that direct adsorption of Cl^−^ on the catalyst accelerates the ClOR process, Lim *et al.* [37] developed carbon nanotubes (CNTs) supported by atomic Pt (Pt_1_/CNT) for ClORs via pyrolysing a Pt-porphyrin precursor. Remarkably, this catalyst gave nearly 100% selectivity for ClORs in acidic media with low Cl^−^ concentrations (0.1 M), as well as in neutral media. *In situ* X-ray absorption spectroscopy (XAS) uncovered that the superior ClOR activity is ascribed to the direct adsorption of Cl^−^ on the Pt−N_4_ sites (Fig. 3d). Compared with dimensional stable anodes, Pt_1_/CNT also demonstrated slightly better activity in naturally neutral seawater. Similarly, Liu *et al.* [38] reported an oxygen-coordinated ruthenium single-atom catalyst (Ru–O_4_ SAM) for chlorine synthesis, which exhibited chlorine selectivity of >98% and stability over 1000 h at 1000 mA cm^−2^. A series of *in operando* characterizations and density functional theory (DFT) calculations revealed that Cl^−^ adsorbs directly onto the surface of Ru atoms of Ru–O_4_ SAM, giving rise to a reduction in the Gibbs free-energy barrier as well as an increase in Cl_2_ selectivity.

Unlike noble-metal-based ClOR catalysts, low-cost metal-based catalyst development is practically valuable for ClORs in which seawater is used as the feedstock [39–43]. A novel methodology is to fabricate materials within a ClOR electrolyte, as demonstrated by Xiao *et al*. [44], who presented a self-adaptive amorphous CoO*_x_*Cl*_y_* catalyst that was *in situ*-deposited on bare F-doped tin oxide in an acidic saline electrolyte containing Co^2+^ and Cl⁻ ions. Online DEMS and rotating ring-disk electrode measurements confirmed that this catalyst exhibited ∼100% chlorine selectivity in a 0.5 M Cl^−^ brine solution. A series of *in situ* characterizations and DFT calculations demonstrated that the *in situ* introduction of Cl^−^ prevents Co sites from reaching a higher oxidation state due to a transition of the active site from Co* to Co–O* for the ClOR, thereby suppressing O–O bond formation to an OER (Fig. 3e). This *in situ* synthesis strategy equipped an as-prepared catalyst (CoO*_x_*Cl*_y_*) with the ability to adapt to real electrochemical conditions, realizing ∼100% chlorine selectivity with an overpotential of ∼0.1 V at 10 mA cm^−2^ over 500 h in 0.5 M NaCl.

### Light hydrocarbon electrooxidation in the presence of Cl^−^

**Figure 4.**
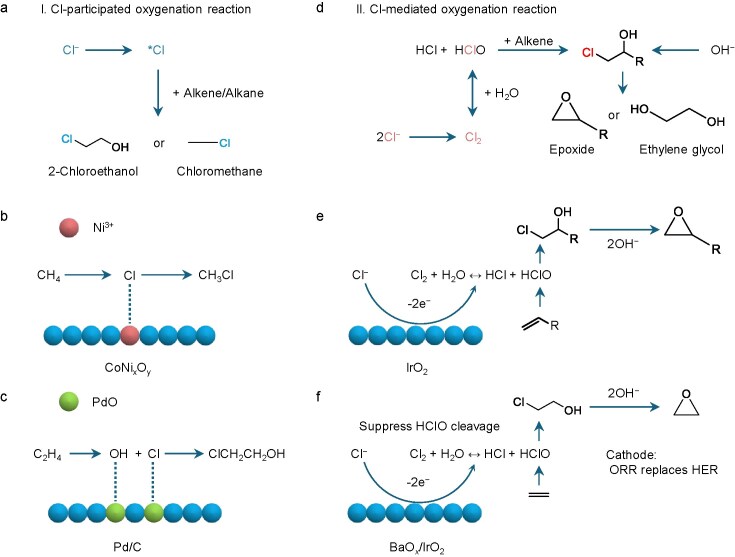
(a) Illustration of Cl-participated oxygenation reaction. (b, c) Schematic works of Cl direct participation of (b) CH_4_ to CH_3_Cl on CoNi*_x_*O*_y_* by chlorine intermediates [55] and (c) C_2_H_4_ to ClCH_2_CH_2_OH on Pd/C by *Cl and *OH [56]. (d) Scenario of Cl-mediated oxygenation reaction. (e, f) Schematic configurations of Cl mediation of (e) alkene oxidation to epoxide on IrO_2_ [46] and (f) ethylene to oxirane on BaO*_x_*/IrO_2_ [60].

The sluggish kinetics of OERs and the corrosive effect of Cl⁻ in seawater electrocatalysis raise significant cost and safety concerns in crude hydrogen production. Accordingly, a paired light hydrocarbon (CH_4_, C_2_H_4_, etc.) electrooxidation reaction with an HER in a Cl^−^-containing electrolyte is a sustainable and meaningful way for chemical transformation and upgrade [45–50]. The anodic oxidation of small molecules offers favorable kinetic and thermodynamic advantages over OERs, enabling higher current densities at lower voltages to produce value-added chemicals [51,52]. It typically can be categorized into two scenarios: a Cl-participated oxygenation reaction and a Cl-mediated oxygenation reaction. The first scenario demonstrates that the Cl^−^ evolves intermediate chlorine species (*Cl) under anodic potentials, which can activate O−H and C−H bonds of alkane or alkene, facilitating their electrooxidation into chloride-containing oxygenates (Fig. 4a). This direct utilization of Cl^−^ in light hydrocarbon electrooxidation reactions can significantly promote both the activity of the catalyst and the selectivity of the target products [53,54]. For example, Wang *et al.* [55] reported that mixed cobalt–nickel spinels enabled the activation and conversion of CH_4_ into CH_3_Cl without overoxidation to CO_2_ (Fig. 4b). Electron paramagnetic resonance (EPR) measurements and DFT calculations revealed that Ni^3+^ at octahedral sites stabilizes *Cl intermediates without the need for plasma, leading to outstanding activity and selectivity of the CH_4_-to-CH_3_Cl transformation. More importantly, the catalyst also demonstrated the capacity for direct methane conversion in simulated seawater with an activity of 86.5 mmol g^−1^ h^−1^ and a CH_3_Cl/CO_2_ selectivity of 7. Furthermore, in acidic seawater, a novel insight involving natural chloride participation suggests that the synergism of *Cl and *OH on the Pd surface can directly activate ethylene to 2-chloroethanol prior to the onset potential of the ClOR, contrasting with above chloride-mediated ethylene oxidation at high potential with Cl_2_ participation (Fig. 4c) [56].

Differing from the direct Cl-participated reaction, the Cl-mediated oxygenation reaction consists of the heterogeneous ClOR followed by a homogeneous alkene oxidation reaction (Fig. 4d), which exhibits much higher activity and selectivity for alkene electrooxidation reactions. This occurs because the indirect reaction pathway relies on the exceptional oxidizing ability of the *in situ* generation of hypochlorous acid (HClO). Especially, as shown in Fig. 4e, Cl_2_ generated at the anode is followed by HClO, which readily oxidizes ethylene to 2-chloroethanol. In the meantime, hydroxyls remain after the HER in the catholyte, which is mixed with 2-chloroethanol to form ethylene oxide [46]. This Cl-mediated ethylene oxidation with IrO_2_ as the anode catalyst reached a current density of 1.0 A cm^−2^ with an oxirane faradaic efficiency (FE) of 60 (±4)%. This strategy presents a breakthrough in preventing ethylene overoxidation and the OER. Additionally, cheaper Co_3_O_4_ catalysts were developed for the electrooxidation of ethylene [57]. Electrochemical kinetic tests and *in situ* electrochemical XAS demonstrated the significance of ethylene in the oxidation reaction, i.e. the vacant cobalt oxide sites shift to chlorine-adsorbed sites, and the rate-determining step changes to a subsequent chemical step in the presence of ethylene, differing from previous ClOR mechanisms. It is verifiable that 2-chloroethanol gave >70% FE in both synthetic and natural seawater, and it was further able to convert to ethylene glycol. Based on this, a CrO*_x_*-decorated IrO_2_ catalyst was designed to achieve ethylene glycol production at the anode [58]. The mechanism shows that the favorable adsorption of *Cl rather than *OH on the CrO*_x_*– IrO_2_ catalysts promotes the ClOR, thereby improving the selectivity of ethylene glycol. Pairing a Cl-mediated ethylene oxidation reaction with an H_2_O_2_-mediated ethylene oxidation reaction in an electrolyser, a co-production of pure ethylene glycol on both electrodes was achieved. More recently, Cai *et al.* [59] crafted a modified Co_3_O_4_ with Ir single atoms (Ir_1_/Co_3_O_4_) that presented efficient ClOR activity for ethylene oxidation to 2-chloroehtanol in neutral seawater. The key idea is that the presence of an atomically asymmetrical Ir–O–Co configuration with strong electron coupling effects in Ir_1_/Co_3_O_4_ promoted both ClOR activity and catalyst stability. An integrated system demonstrated the anodic chloride-mediated and cathodic H_2_O_2_-mediated ethylene oxidation reactions to produce 2-chloroehtanol and ethylene glycol, respectively, presenting a total FE of ∼170%. However, the cleavage of HClO leads to unreactive active chlorine in this mediated process, which results in a loss of FE for ethylene oxide and requires high energy input. Li *et al.* [60] thus introduced BaO*_x_*/IrO_2_ hybrid catalysts that achieved an ethylene-to-oxirane FE of 85%–91% at 100–1500 mA cm^−2^. *In situ* Raman measurements and DFT calculations evidenced that a Ba–O–Cl structure formed on the BaO*_x_*/IrO_2_ surface and suppressed the cleavage of hypochlorous acid (Fig. 4f). The HER at the cathode was replaced by an oxygen reduction reaction to reduce the energy input (5.3 MJ per kg of ethylene oxide). Also, bromine in seawater offers potential benefits for the oxidation of light hydrocarbon, as the *in situ* generation of HBrO can oxidize ethylene or propylene to produce bromohydrin (2-bromoethanol or 3-Bromo-1-propanol), which can then be post-treated to target products such as ethylene oxide, propylene oxide or triethanolamine [61–63].

The alternative production of chlorinated chemicals via seawater electrocatalysis is an attractive and potentially sustainable strategy. However, a comprehensive techno-economic assessment is essential to evaluate its practical feasibility. Factors such as the production pathway, downstream conversion, separation process and purity levels must be critically considered, as they directly integrate with the overall cost and scalability. Comparison of the chloride-involved reactions and established industrial route is indeed meaningful, as the potential production and purification costs of chlorinated chemicals in the electrochemical route may approach or even exceed their corresponding market value. Consequently, the advances of an optimized integrated process design (encompassing both reaction efficiency and cost-effective separation) are the premises to achieving overall profitability.

### Specific adsorption of Cl^−^ for carbon-dioxide-reduction reaction

**Figure 5.**
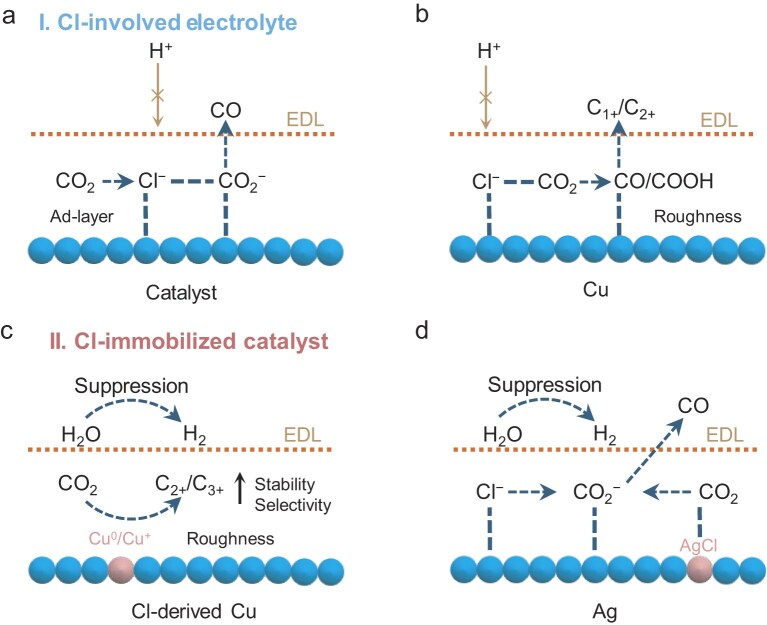
Strategies toward Cl^−^ utilization of seawater in CRR, which accounts for Cl^−^ adsorption on (a) Zn, Ag or Au catalyst and (b) Cu for enhancing CRR performance in Cl-involved electrolyte. Cl-immobilized catalysts for promoting CRR selectivity and stability by (c) creating Cu^0^/Cu^+^ and (d) AgCl active sites.

The influence of Cl^−^ in seawater on the carbon-dioxide-reduction reaction (CRR) was recognized for its ability to reduce overpotential, reconstruct catalyst morphology and stabilize intermediates. In this section, we clarify two strategies for enhancing CRR towards high value of C_1+_/C_2+_/C_3+_ products by modifying the solution (electrolyte) or solid (catalyst) interfaces. These strategies include Cl-involved electrolytes and Cl-immobilized catalysts. On the solution side, abundant Cl^−^ is available to absorb onto a small number of charged metal catalyst (Zn, Ag or Au) surfaces to establish an adsorption layer (ad-layer, Fig. 5a). This typical ad-layer effectively suppresses HERs by hindering the proton approach but promotes electron transfer via the metal−Cl ad-layer to reduce the activation barrier of the CO_2_ molecule, thereby benefitting the formation and stabilization of *CO_2_^−^ intermediates toward carbon oxide (CO) formation [64–66]. Similarly, this specific adsorption with high Cl^−^ coverage also roughens the copper (Cu)/oxidized copper electrode as well as reducing the overpotential of the CRR. The presence of Cl^−^ in the electrolyte positively affects both the thermodynamics and kinetics of the CRR on the Cu electrode and tunes the selectivity of the CRR products. As shown in Fig. 5b, although Cl^−^ leads to morphological changes in the Cu surface, the improvement in the CRR performance primarily results from the adsorption of Cl^−^ on the Cu surface or Cu(I) sites. The special adsorption modulates the local electronic structure of the Cu surface, which makes the localized Cu surface negatively charged. The partial negative charge from the Cl^−^ to the CO_2_ not only lowers the free energy of the key intermediates (*CO/*COOH), but also stabilizes them through both electrostatic interaction and electronic effects [67–70], thereby energetically favoring its retention and subsequent reduction to CO, hydrocarbons (such as CH_4_ and C_2_H_4_) and alcohols. For example, Yang *et al.* [69] reported that a dual-phase copper catalyst with abundant Cu(I) sites at the amorphous−nanocrystalline interfaces can achieve a high C_2+_ product FE of 80% in a chloride-contained electrolyte at a partial current density of 322 mA cm^−2^. It was found that the Cl^−^ was adsorbed onto the Cu(I) sites of the catalysts and thereby improved the local *CO coverage for C−C coupling.

Furthermore, the development of Cl-immobilized catalysts represents a significant advancement of the solid side of CRRs. Cu catalysts containing Cl^−^ have been shown to tune CRR selectivity and improve CRR stability. The oxidative etching effect of Cl^−^ on these Cu catalysts results in substantial changes to the catalyst surface, leading to roughness morphology with increased defect sites. The morphological effect of chloride-derived Cu can tune the CRR product selectivity. It also accompanies the Cu^0^/Cu^+^ species formation and Cl^−^ adsorption, which boosts C−C coupling and in turn induces C_2+_/C_3+_ product formation (Fig. 5c) [71–77]. Besides, Cl^−^ in seawater also demonstrates the potential advantages of CO generation over silver (Ag) or gold (Au) catalysts [78,79]. For example, Cl^−^ adsorption onto an Ag catalyst can promote *CO and *COOH intermediates during the CO_2_-to-CO conversion [77,78,80]. In addition, the derived AgCl species under cathodic bias leads to enhanced CRR performance. For instance, because of surface Cl^−^ modification over Ag catalysts (AgCl nanoparticles) through *in situ* electroreduction bias, the strong interaction of AgCl can enhance CO_2_ capture and promote *CO_2_^–^ intermediate formation (Fig. 5d) [81,82]. This approach achieves high selectivity of the CO_2_-to-CO conversion under near-neutral conditions. Despite the rapid advancements of *in situ* characterization techniques providing direct information for understanding the complex CRR mechanisms, there are few reports using these methods to elucidate the promotion effect of Cl^−^ on CRR performance. This has led to inadequate recognition of the actual effects of Cl^−^ on CRR performance, including its role in promoting the adsorption of reaction intermediates for enhanced CRR activity and its influence at the solution–solid interface.

### Other Cl^−^-involved reactions

**Figure 6.**
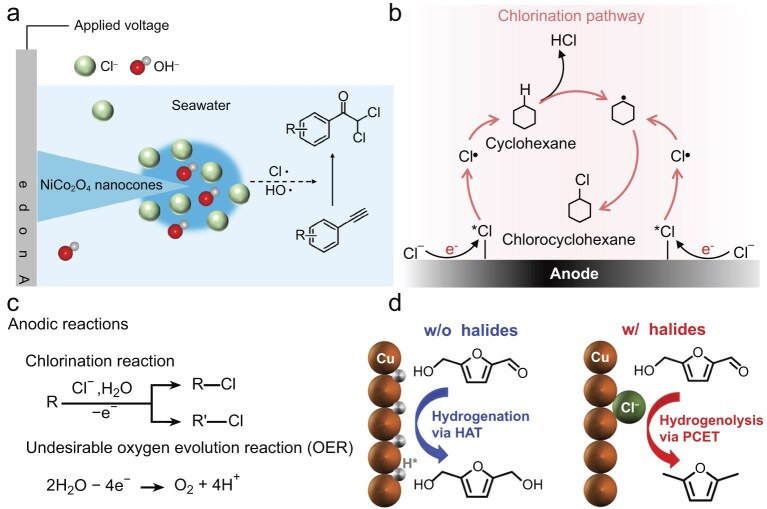
Other Cl^−^-involved reactions. (a) Schematic diagram of alkynes electrooxidation to α,α-dichloroketones in seawater. Reproduced with permission from [83]. Copyright 2024, American Chemical Society. (b) Cl· radical-mediated C(sp^3^)−H bond chlorination pathway for cyclohexane. Reproduced with permission from [84]. Copyright 2023, Springer Nature. (c) Selective anodic chlorination reactions and side OER. Reproduced with permission from [87]. Copyright 2025, American Chemical Society. (d) Proposed reaction pathways for the hydrogenolysis and hydrogenation of 5-hydroxymethylfurfural. HAT: hydrogen atom transfer; PCET: proton-coupled electron transfer. Reproduced with permission from [89]. Copyright 2023, American Chemical Society.

Cl^−^ directly influences electrocatalytic performance in other Cl^−^-involved reactions that have potential applications in seawater electrocatalysis. In one scenario, Cl^−^ directly participates in reactions via Cl· radicals during the electrooxidation process. For instance, in the synthesis of *α,α*-dichloroketone, an FE of 61% was obtained over NiCo_2_O_4_ nanocones through the addition of Cl· and OH· radicals to 1-chloro-2-ethynylbenzene in seawater (Fig. 6a) [83]. Moreover, Cl· radicals can activate inert C(sp^3^)−H bonds. This approach enables the chlorination of cyclohexane to chlorocyclohexane over Pt/IrO*_x_*, while significantly suppressing chlorine formation (Fig. 6b) [84]. An FE of 93.8% toward chlorocyclohexane was achieved in concentrated seawater at a current of 1 A. Following a similar concept, ethanol can undergo electrochlorination to 2-chloroethanol over boron-doped diamond or tetrahedral amorphous carbon nitride catalysts [85]. Another noteworthy Cl^−^-involved reaction is that adsorbed Cl can couple with urea during urine electrooxidation to form *N*-chlorourea intermediates for N_2_ generation [86].

In another scenario, Cl^−^ participates in anodic reactions but forms different Cl-containing products. The side reaction evolves to an OER due to the battle between the Cl· and OH· radicals (Fig. 6c) [87]. Through introducing an anion (ClO_4_^−^) to widen the anodic electrical double layer (EDL) or tuning the interfacial hydrogen-bond of water (H_2_O/dimethyl carbonate hybrid electrolyte), a higher selectivity of Cl-containing target products were obtained via suppressing the OER in seawater [87,88]. Admittedly, Cl^−^ also has a positive influence on the product selectivity of hydrogenation reactions. For example, Cl^−^ adsorption over a Cu surface significantly decreases surface proton coverage during 5-hydroxymethylfurfural reduction (Fig. 6d) [89]. Thus, Cl^−^ coverages are achieved to enhance the selectivity of 2,5-dimethylfuran by limiting 2,5-bis(hydroxymethyl)furan formation.

Finally, we present a summary Table 2 of the reported key chloride-related mechanisms in the various electrochemical reactions in seawater. These mechanisms cover adsorption, participation, immobilization, synergistic mediation and electrolyte engineering, with their corresponding effects on performance concisely outlined to complement the detailed discussions above.

**Table 2.** Summary of key chloride-utilization mechanisms and their impacts on electrocatalytic performance.

Mechanism type	Representative strategy	Example catalyst/reaction system	Role of Cl^−^	Impact on performance
Adsorption	Surface binding of Cl^−^ to active sites	Pt, RuO_2_, NiFe-LDH, Cu	Alters surface electronic structure	Enhances activity and stability of catalyst; tunes selectivity for desired products
Participation	Direct involvement of Cl^−^ in reaction pathway	Cl-mediated ethylene/propylene oxidation; Cl-assisted OER	Cl^−^ forms active intermediates (e.g. HClO, Cl_2_); participates in reaction directly	Enables unique reaction pathways
Immobilization	Anchoring Cl^−^ within catalyst lattice or porous host	CoO*_x_*Cl*_y_*, Ag, Co_2_(OH)_3_Cl	Provides stable local Cl^−^ concentration near active sites	Improves catalyst stability
Synergistic mediation	Coupling Cl^−^ utilization with other ions	Pd, NiCo_2_O_4_	Cl^−^ works in concert with other species to modulate catalytic environment	Tunes product selectivity
Electrolyte engineering	Optimizing Cl^−^ concentration or source	Acidic seawater, concentrated seawater	Controls Cl^−^ activity and competing side reactions	Balances Cl^−^ benefits with corrosion minimization

## RATIONAL UTILIZATION OF CATIONS

The dominating Na^+^ in seawater holds potential application prospects for seawater electrocatalysis [5,10], yet the down-ordered cations (Mg^2+^ and Ca^2+^) receive less attention because contemporary works have mainly focused on alkaline-natural seawater electrocatalysis (alkaline additives) or simulated seawater (an alkaline electrolyte with NaCl). If natural seawater is used, the addition of alkali (e.g. KOH, $800 tonnes^−1^) or buffer is recommended to refine the seawater as the electrolyte [13,90]. This procedure increases the initial cost input and pre-removes Ca^2+^ and Mg^2+^ cations via forms of hydroxides. In this way, seawater electrocatalysis is trapped by Cl^−^ corrosion and makes the researchers merely concerned about the development of anode materials to obviate Cl^−^ attack. It should be noted that Mg(OH)_2_ is deemed to be a high-value-added chemical due to the large demand in various fields [91]. An intended design for a continuous Mg(OH)_2_-recovery system from seawater presents a sustainable route to recycle resources based on seawater electrocatalysis. More and more researchers have been aware of the significant difference between simulated seawater and natural seawater. Both Cl^–^ corrosion and Ca^2+^ and Mg^2+^ precipitation should be considered for the real seawater electrocatalysis.

### The utilization of Na^+^

**Figure 7.**
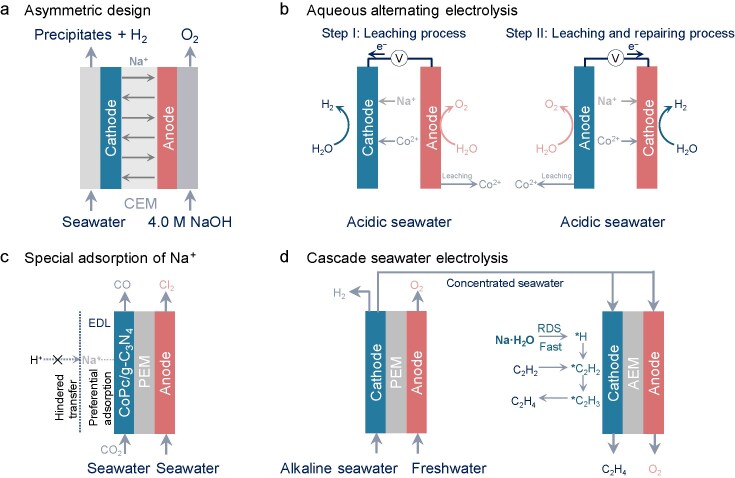
Rational designs of Na^+^ utilization towards (a) asymmetric design in direct seawater electrolysis, (b) aqueous alternating electrolysis in acidic seawater, (c) special adsorption of Na^+^ in CRR and (d) cascade seawater electrolysis for HER and acetylene hydrogenation reaction.

The Na^+^ in seawater with favorable conductivity for electrocatalysis is ∼0.5 M. This has spurred the innovation in membranes with Na^+^ utilization that show significantly promotional roles in seawater electrocatalysis. Shi *et al.* [92] devised a Na^+^-conducted asymmetrically flowing electrolyser that prevents the Cl^−^ in seawater from crossing to the anode (NaCl solution or natural seawater), avoiding Cl^–^ corrosion at the anode and alleviating Ca^2+^ and Mg^2+^ precipitates (pushed away by the flow electrolyte) at the cathode in natural seawater (Fig. 7a). With this pH-asymmetric electrolyser design, current densities of 10 and 100 mA cm^−2^ only required a cell voltage of 1.31 and 1.46 V, respectively. Thus, decoupling of the anolyte and catholyte can effectively reduce the energy input for hydrogen production. It was reported similarly by Ren *et al*. [93] that a monovalent-selective cation exchange membrane (CEM) was fabricated to separate seawater from concentrated NaOH. The CEM remarkably prevents the penetration of the Mg^2+^, Ca^2+^ and Cl^−^ ions in seawater into NaOH chambers without influencing the Na^+^ and water migration for seawater electrocatalysis. Given the Na^+^ conductivity and harmful ClOR in seawater electrocatalysis, applying for advanced electrooxidation reactions to replace the OER/ClOR at the anode also offers a lower cell potential to yield hydrogen and realizes chlorine-free electrocatalysis. Therefore, Zhang *et al*. [94] utilized a CEM for the Na^+^ transport between anodic and cathodic chambers and restricted chloride migration to the anode. Connecting S^2−^ pollutant degradation at the anode with alkaline seawater reduction at the cathode saved energy input for hydrogen production. It was found that a low cell voltage of 0.97 V was obtained at 300 mA cm^−2^ with 50% lower energy input compared with alkaline seawater electrocatalysis.

Unlike the asymmetric design, Liang *et al*. [95] employed an aqueous alternating electrocatalysis by deliberately making the electrode outer layer dissolve freely and periodically repaired/fixed this protective layer, as demonstrated in Fig. 7b, in which Ni-based electrodes doped with Fe-group ions (Fe²⁺, Co²⁺, Ni²⁺ and Mn²⁺) were used. The cathode initially occurs in the HER and deposition of metal ions, while O_2_ evolves with the leaching-out of metal ions over a metallic anode (Step I). The cathode and anode reverse after a period in the outer layer of the electrode to improve the stability of the anode in acidic seawater. This approach involved electrocatalysis to repeat the above-mentioned deposition–dissolution processes (Step II). This aqueous alternating electrocatalysis enabled prolonged operation at ampere-level current densities for 93.8 h in an acidic environment. The synergistic interaction between Co²⁺, Na⁺ and other alkaline metal cations plays a critical role in extending electrode lifespan. More details for the longer electrode survival are divided into three aspects: synergistic effects of hydrated Na^+^ (Na·H_2_O), moderate interactions between Na^+^ and metal Co, and suitable adsorption strengths of metal Co. Analogous to the special adsorption of Na^+^ on the catalyst, Tan *et al*. [96] prepared a CoPc/g–C_3_N_4_ electrocatalyst with a negatively charged surface that preferentially adsorbed Na^+^ ions (Fig. 7c). This adsorption capacity of Na^+^ on CoPc/g–C_3_N_4_ significantly impeded H^+^ transfer and enhanced the appreciable 89.5% FE of CO with a current density of 16.0 mA cm^−2^ in natural seawater and 25 h of operation stability in simulated seawater. *Ex situ* experiments showed that, by replacing the ClOR with the OER at the anode, the rapidly decreased pH value of the anolyte also improved the CRR performance. This integrated design of the co-production of CO and Cl_2_ can achieve a 98.1% FE of CO at a cell voltage of 3 V in simulated seawater.

In addition to the selective adsorption of Na^+^ on catalysts, Na^+^ ions are beneficial in interfacial water dissociation [97]. In natural seawater, Na^+^ can concentrate on the Cu_3–_*_x_*Co*_x_*P catalyst, enhancing HER performance and suppressing precipitation formation [98]. *In situ* fourier transform infrared (FTIR) spectra reveal that the O–H stretching mode of interfacial water (ν_O–H_) on the Cu_3–_*_x_*Co*_x_*P exhibits a slight blue shift as the applied potential decreases. Coupled with *in situ* Raman spectra analysis, this indicates that the proportion of Na·H_2_O species with weak hydrogen-bond interactions on Cu_3–_*_x_*Co*_x_*P facilitates the water splitting. Following this principle, the use of concentrated seawater from alkaline seawater electrocatalysis as the electrolyte enabled electrocatalytic acetylene hydrogenation to ethylene, achieving a 71.2% FE of ethylene at a large partial current density of 1.0 A cm^−2^ [99]. A set of complementary Raman and FTIR spectroscopy measurements and controlled electrochemical tests evidenced that Na^+^ cations in concentrated seawater dynamically promoted the unsaturated interfacial water (Na·H_2_O) dissociation to provide protons for acetylene hydrogenation (Fig. 7d). The integrated system of alkaline seawater electrocatalysis and acetylene hydrogenation reaction exhibited a stability of 32 h for the co-production of hydrogen and ethylene at 400 mA cm^−2^.

### Mg hydroxide recovery

**Figure 8.**
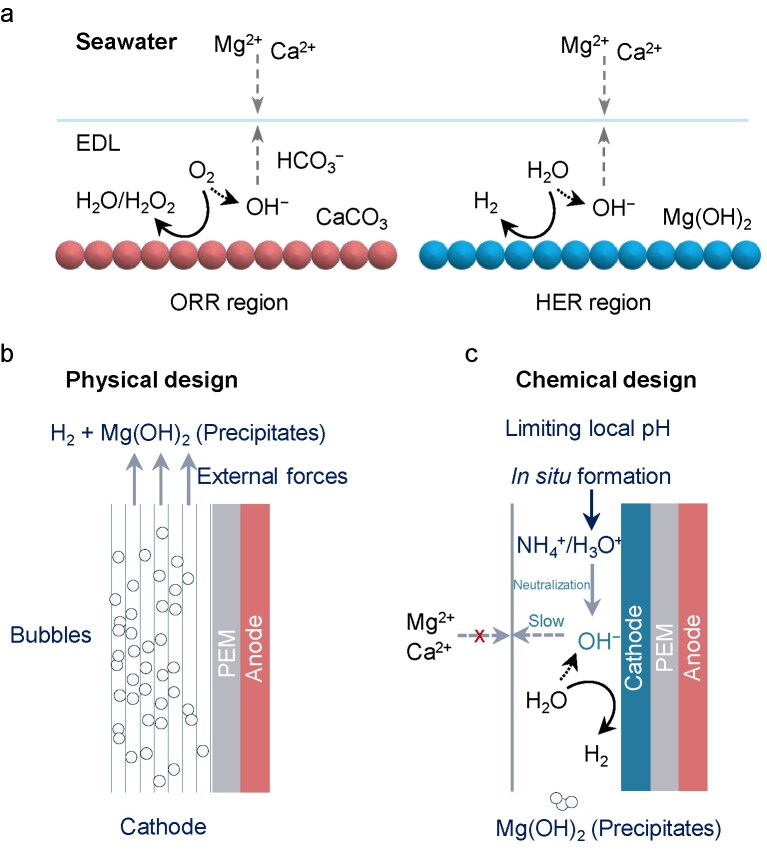
(a) Electrodeposition of minerals in seawater in ORR and HER regions. Schematic configuration of the co-production of H_2_ and Mg(OH)_2_ in natural seawater by (b) physical and (c) chemical designs.

The ions in seawater are unable to keep the local pH near the cathode stable during the HER process. Therefore, rapid consumption of protons causes the local pH to increase significantly, thereby resulting in the precipitation of Mg^2+^ and Ca^2+^ hydroxides. Given the high value of Mg(OH)_2_, modifications to the physical and chemical properties of the catalysts are anticipated to disrupt the local pH gradient, producing Mg(OH)_2_ nanoparticles and enhancing H_2_ activity and stability at the cathode. However, the Ca^2+^ in seawater can affect the purity of Mg(OH)_2_ during the electrochemical reduction process. A recent study proposed a schematic of the active potential region at the cathode toward the electrodeposition of minerals in seawater that involved an oxygen reduction reaction (ORR) and a water reduction reaction (Fig. 8a) [100]. At applied potentials of −0.8 and −1.0 V_Ag/AgCl_ toward the ORR region, the electrodeposition process is limited by the oxygen mass transport in seawater. Aragonite and calcite (CaCO_3_) favor continuously growing on the cathode surface, suppressing oxygen transfer as well as reaction sites. At applied potentials of −1.2 and −1.6 V_Ag/AgCl_ toward the HER region, the limiting factor is the kinetics of mineral formation. Brucite is the dominant precipitate on the cathode surface. In addition, the electrodeposits in the above two regions continuously grow on the electrode surface instead of forming in bulk. But, in the HER region, the precipitates (Mg(OH)_2_) typically detach from the cathode surface at a higher applied potential and Ca(OH)_2_ is detected on the cathode; it is reported that the calcareous deposit has more structural strength with time due to the ‘hard’ properties of calcium carbonate [11,101].

To alleviate Mg^2+^ and Ca^2+^ precipitation and recover the value-added Mg(OH)_2_, Liang *et al.* [102] developed a self-cleaning Pt/carbon cathode for the onsite co-synthesis of H_2_ and Mg(OH)_2_ in natural seawater (Fig. 8b, Physical design). This physical design introduced external flow pipes to expedite autonomously moving gas bubbles/seawater (fluid) flows for repelling precipitates [92]. This Pt/carbon electrode is capable of operating at a current density of 170 mA cm^−2^ for ≥48 h and achieving a Mg(OH)_2_ formation rate of 0.345 g h^−1^ cm^−2^. In a fairly similar design, Liang *et al.* [103] also fabricated a honeycomb-type 3D structure cathode with rich of longitudinally arranged/elongated microchannels for anti-precipitates to adhere to in natural seawater electrocatalysis. In principle, this porous carbon framework releases large quantities of small-sized H_2_ bubbles to give buoyancy and external forces to clean precipitates at the cathode surface compared with disordered pore structures such as that of nickel foam. The FE of H_2_ was close to 100% for a flow-type electrolyser with this optimal cathode operated at an industrial-relevant current density of 500 mA cm^−2^ for 150 h in natural seawater. Likewise, aiming for the co-electrosynthesis of H_2_ and Mg(OH)_2_ in seawater electrocatalysis, electrode surface modification inhibits Mg^2+^ and Ca^2+^ precipitate adherence [104]. Yi *et al.* [14] constructed a Pt-coated NiCu alloy with a solidophobic surface property, achieving an unprecedented lifespan of >1000 h at 100 mA cm^−2^ in a simulated electrolyte with Mg^2+^ and Ca^2+^ ions. This especially solidophobic behavior contributes to the adsorption of surface water and thereby enforces Mg(OH)_2_ nucleation homogeneously in the electrolyte, avoiding the blockage of precipitates on its surface. As a result, this electrode is able to produce H_2_ evolution and high-purity Mg(OH)_2_ simultaneously. To effectively extract the solid Mg(OH)_2_, an innovative electrolyser was devised by optimizing the channel structure of the polar plates. Their efforts yielded a substantial collection of 19.07 g of Mg(OH)_2_ with a purity of >99.0% after operating for >100 h at a current density of 100 mA cm^−2^ in 20 L of natural seawater, all while continuously evolving H_2_. The physical design of the electrode endows it with physically external forces to push away precipitates with the co-production of H_2_ and Mg(OH)_2_. Conversely, the chemical design of the electrode focuses on enhancing HER performance and limiting the local pH increase with anti-precipitation capacity. As substantial OH^−^ is left over on the cathode after the HER, the alkaline Earth metal ions would react with this OH^−^ and precipitate on the cathode when the local pH over the catalyst is >9.5. Therefore, the research target is to sweep the OH^−^ to avoid precipitation coverage and in turn refresh the active sites of the HER. Bao *et al.* [11] reported that introducing a local acid-like environment onto the surface of the Pt/WO₂ catalysts during the HER effectively suppressed precipitate formation. The *in situ*-formed hydrogen tungsten bronze (H*_x_*WO*_y_*) phase during the HER process was confirmed as a proton reservoir to supply H_3_O^+^, which can neutralize OH^−^ for precipitation formations (Fig. 8c). As a result, this catalyst operated at 100 mA cm^−2^ for >500 h in direct seawater electrocatalysis. Based on the design of a flow-type electrolyser using Na⁺ conductivity and chloride-free electrocatalytic principles, a cell potential of 2.47 V was achieved at 500 mA cm^−2^ for 100 h in natural seawater. Another breakthrough study was reported by Zhang *et al.* [105] in which *in situ*-generated ammonium groups (NH_4_^+^) at the Mo_2_N surface can be hydrogen-bonded with OH^−^ to suppress precipitation formation and enhance the HER. Consequently, a flow-type electrolyser assembled with this catalyst gave a current density of 1 A cm^−2^ at 1.87 V and 60°C for >900 h of stability in simulated seawater.

The current strategies for high-purity Mg(OH)_2_ recovery rely on the additional flow force or flow-through skeleton support electrode, which can sweep away precipitates to release active sites. The period separation operation is needed to refresh the electrolyte; though it is fundamental for high-purity Mg(OH)_2_ recovery, it has been relatively underexploited. Furthermore, the proton exchange membrane (PEM) in natural seawater electrolysis is fairly strategic. Unlike the AEM, the PEM transports protons rather than Cl^−^, suppressing the chloride crossover to mitigate Cl^−^ corrosion. Additionally, the PEM shows superior stability and reduces the susceptibility to scaling potential, making it more favorable than AEM for long-term seawater electrocatalysis operations. Although the economic feasibility of direct seawater electrocatalysis has been demonstrated through catalyst advances to co-produce H_2_ and Mg(OH)_2_ at the cathode [14,98,102], no research has been dedicated to elucidating the potential mechanism for pure Mg(OH)_2_ formation in the presence of Ca^2+^ and other seawater constituents. Understanding the selective precipitation process is critically important, as it not only would deepen the fundamental insight into seawater electrocatalysis chemistry, but also enable the rational design of integrated systems for the co-production of H_2_ and Mg(OH)_2_. Besides, advancing electrolyser technology to enable the steady collection of Mg(OH)_2_ without intermittent start–stop cycles remains a critical challenge.

## RARE-METAL EXTRACTION FROM SEAWATER

The ocean contains various rare-metal ions, such as beryllium, lithium, rubidium and gallium. Even though the concentrations of these ions are relatively low, indispensable applications in various fields make their recovery highly desirable. However, extracting these ions from high-salinity seawater is challenging. Conventional physicochemical adsorption methods use charged material to attract ions though coulomb forces, but slow adsorption rates and competition from other ions lower the efficiency and selectivity. Electrochemical extraction strategies present various advantages over conventional adsorption methods, including low energy input, eco-friendly, long lifespan and easy regeneration. This section discusses the representative uranium and lithium extraction from seawater regarding electrochemical extraction methods and cell designs.

### Uranium extraction from seawater

Uranium is crucial for nuclear fuel, which provides large-scale electricity generation without greenhouse gas emissions. With rising demand, uranium resources in terrestrial ores (∼6.3 million tons) have an emerging shortage due to the increasing consumption rate, while seawater holds an estimated 4.5 billion tons of uranium [106,107]. However, despite this abundance, its low concentration (∼3.0 ppb) and high salinity make efficient extraction difficult [108]. To address the problems, current representative studies of uranium extraction from seawater have been developed, such as electrodeposition, electrosorption and electrocatalysis processes. The core concept of those methodologies is that the diffusion of uranyl ions can be accelerated by an electric field that breaks the thermodynamic adsorption–desorption equilibria in seawater to enhance the mass transfer diffusion of uranium.

**Figure 9.**
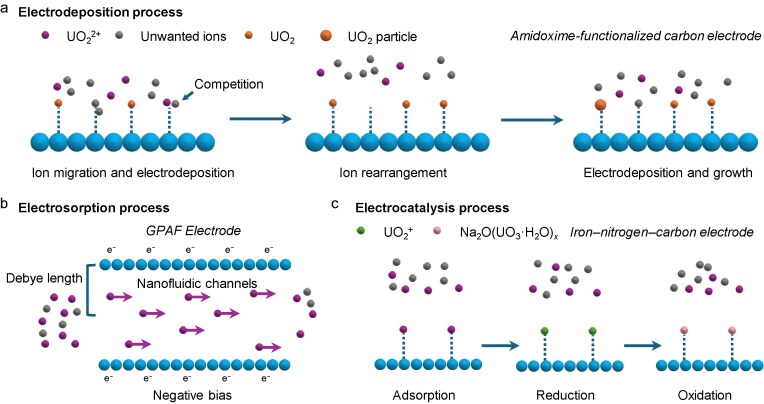
Schematic processes of uranium extraction from seawater. (a) Electrodeposition process [109], (b) electrosorption process [117] and (c) electrocatalysis process [119].

The electrodeposition process undergoes selective electrosorption and *in situ* deposition as well as the growth of uranyl. Liu *et al.* [109,110] employed a half-wave rectified alternating current electrochemical (HW-ACE) method with an amidoxime-functionalized carbon electrode to extract uranium from seawater under a voltage alternating between −5 and 0 V (Fig. 9a). This HW-ACE method includes five steps: uranyl ions were captured, reduced and deposited as UO_2_ particles, and periodic repeat of the operation leads to a continuous growth of UO_2_ and improved uranium extraction. This approach extracted 1.62 μg of uranium from 4 L of unspiked seawater and was later demonstrated on chitosan-functionalized electrodes and S-terminated MoS_2_ nanosheets [111,112]. A dual-function strategy was also proposed to achieve either uranium extraction or HER in seawater [113,114].

Although the electrodeposition process has evidenced high capacity and faster kinetics in uranium extraction from seawater, the concentration of other ions, at several orders of magnitude higher than that of uranyl ions in seawater, significantly diminishes the uranium-extraction efficiency. Capacitive deionization utilizes the electrosorption of charged ions in the EDL layer for water treatment. Ismail and Yim [115] first applied the electrosorption process for uranyl-ion extraction from seawater in 2015. This approach contributes to the selectivity of electrosorption to the differences in the charge density of ions. However, the thickness of the EDL layer or a Debye length of <1 nm limits the electrode activity for uranyl-ion extraction [116]. Thus, Wang *et al.* [117] developed graphene-based porous aromatic framework electrodes with nanofluidic channels tailored for uranyl ions at twice the length of the geometric dimension of a UO_2_^2+^ ion (a maximum length of 6.04−6.84 Å) (Fig. 9b). Creating a charge-regulated region under negative bias allows selective ion transport by repelling co-ions and enhancing cation mobility through unipolar transport, achieving ∼16 mg g^−1^ of uranyl adsorption over 56 days. Zhang *et al.* [118] proposed that, differing from the prebuilt absorption sites in materials, the C=O groups on the covalent organic framework (COF) channel can evolve to adjacent phenol–oxygen anions with the coordination atoms (O and N) for rapid uranium extraction from natural seawater; the adsorption rate of the uranyl ions can deliver 4.2 mg g^−1^ d^−1^.

After the electrosorption process, anchored uranyl ions can then undergo the electrocatalysis process for efficient uranium extraction from seawater. For instance, the regenerable redox reaction of Fe(II)/Fe(III) has been demonstrated to extract uranium through U(VI) precipitation, as shown in Fig. 9c [119,120]. This approach involves U(VI)O_2_^2+^ initially being electro-adsorbed by Fe–OH under an electric field. Subsequently, the Fe(0) and Fe(II) reduce U(VI)O_2_^2+^, generating Fe(III) and U(IV). Finally, a negative bias regenerates Fe(III) back to Fe(II), allowing a continuous redox cycle for sustained uranium extraction from seawater. Building on this concept, electrocatalysis for uranyl-ion extraction with the rational catalyst design has emerged as a promising approach to generate U(VI) precipitates [121–125]. For example, Chen *et al.* [122] constructed amidoxime-group-modified porous aromatic frameworks (PAF-144-AO) on flexible carbon cloths that selectively capture uranyl ions and convert them into Na_2_O(UO_3_·H_2_O)*_x_* precipitates in the presence of Na^+^ under an alternating electric field. An extraction capacity of 12.6 mg g^−1^ over 24 days was found in natural seawater.

### Electrochemical lithium recovery from seawater

The growing demand for lithium, driven by the rise in electric vehicles and renewable energy-storage systems, has increased concerns about lithium recovery from unconventional sources. Electrochemical lithium recovery has gained significant attention due to its advantages over passive adsorption processes, such as lower chemical usage and higher time efficiency. Unlike uranium extraction from seawater, which relies on a non-faradaic process that stores uranyl ions in the EDL of electrodes, lithium recovery follows a faradaic process in which ions or electronic charges move across the EDL to interface between the electrode and the electrolyte. This allows the acceptance or donation of electrons at the electrode surface, enabling the recovery of lithium. The selection of the electrode material is crucial in electrochemical lithium-recovery systems and optimizing the electrochemical cell—divided into membrane-free and membrane cells—has become a key focus for improving system performance. In summary, understanding the different types of electrodes and strategies for optimizing electrochemical cells is essential for advancing lithium-recovery technologies. The following section provides an overview of typical electrode materials and cell-optimization strategies in electrochemical lithium recovery.

**Figure 10.**
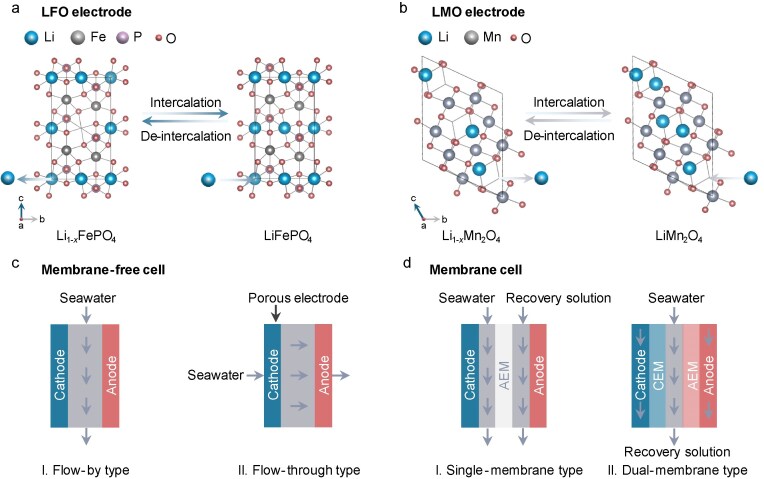
Schematic diagrams of Li^+^ intercalation and deintercalation of (a) LFO electrode and (b) LMO electrode. Electrochemical cell configurations for Li^+^ recovery from seawater. (c) Membrane-free cell, including flow-by type and flow-through type. (d) Membrane cell, including single-membrane type and dual-membrane type.

We list two main types of electrodes for lithium recovery from seawater. The first is lithium iron phosphate (LiFePO_4_; LFO, Fig. 10a)—a polyanionic material favored for its low cost, long cycle life and good stability [126,127]. Although sodium (Na⁺) is the primary cation in seawater and a strong competitor, the olivine structure of FePO_4_ allows Li⁺ to migrate preferentially due to its thermodynamic advantage [128]. Additionally, the lower hydration enthalpy of Na^+^ prevents the intercalation of Mg^2+^ and Ca^2+^ into the FePO_4_ host lattice, allowing selective Li⁺ intercalation into the crystal lattice [129]. Pasta *et al.* [130] used an LFO cathode for lithium capture and a silver anode for chloride recovery, converting a sodium-rich brine covert into a lithium-rich solution (from 1/100 to 5/1). However, LFO electrodes inherit poor ionic diffusion and electronic conductivity due to its 1D olivine structure (1D channel structure) [131]. To address those problems, surface coatings and structural modifications, such as hydrophilic polydopamine, can improve conductivity, wettability and ion mobility, enhancing Li⁺ intercalation. This approach achieved a Li⁺/Na⁺ selectivity of 4330 times in seawater [132,133]. Additionally, Liu *et al.* [134] demonstrated a pulsed electrochemical intercalation strategy by using TiO_2_-coated FePO_4_ electrodes, which promoted the crystal structure stability of the LFO electrode and achieved a selectivity of 1.8 × 10^4^ over 10 cycles of Li^+^ recovery.

The second is manganese oxide (LiMn₂O₄; LMO, Fig. 10b), which offers faster Li⁺ transport and less Na⁺ or Mg²⁺ co-intercalation due to its spinel structure, which facilitates Li⁺ diffusion through Mn^IV^/Mn^III^ transformations [135,136]. Early investigation focused on the nanoscale engineering of LMO materials to enhance the Li^+^-recovery capacity [137–139]. However, LMO experiences capacity loss after 100 cycles [138]. To address this, doping and surface coating with binders or conductive additives were designed to promote LMO electrode stability and enhance Li^+^-recovery selectivity [136,140–145]. For example, Shang *et al.* [145] fabricated a CNT-strung LiMn_2_O_4_ (CNT-s-LMO) electrode, demonstrating a ∼84% Li^+^-extraction percentage in brine and 90% capacity retention as well as negligible Mn loss over 100 cycles. Despite these improvements, the low concentration of Li⁺ in seawater remains a challenge, prompting the development of advanced electrolysers for more efficient extraction.

Li^+^ extraction from seawater was initially implemented in a traditional cell [132,139,140]; subsequently, a flow-type cell was introduced with an additional electrolyte channel parallel to the electrodes, which were composed of seawater, cathode and anode without a membrane (Fig. 10c; I. Flow-by type). This design significantly recovers Li⁺ but is limited by long processing times, diffusion and fewer active sites [146–148]. To rectify these drawbacks, a ‘flow-through type’ was created with a freed stream (seawater) directly through the porous electrode (Fig. 10c; II. Flow-through type) [146,149,150]. The porous materials (microporous carbon particles, active carbon cloth and so on) were assembled as electrodes, which could enhance the removal of salt and hinder the desalination timescale [149]. However, the repeated desalination cycles degraded the electrodes and the continuous production of Li^+^ in the independent cell was impossible [151]. Accordingly, membrane-contained cell was newly proposed to avoid the complicated operation of the single-channel flow cell (Fig. 10d; I. Single-membrane type). This deliberate design realized the continuous recovery of Li^+^ from seawater by introducing a two-channel flow chamber for extracting and concentrating the Li^+^ simultaneously [152]. In a typical recovery process (discharging), the Li-deficient electrode (anode) in the seawater captures Li^+^, which traverses the AEM and concentrates in the anode chamber to form an Li-rich electrode—the recovery solution [153]. To further enhance the recovery capacity, the use of flowable particles rather than static electrodes as the Li^+^-adsorption materials can realize constant ion removal by refreshing the flow electrode (Fig. 10d; II. Dual-membrane type). The flowable particles first capture Li^+^ in the electrode and then release Li^+^ after ion membrane exchange. Thus, the concentrated Li^+^ solution can be further purified in another configuration without disturbing the operation of the recovery set-up. The Li^+^ extraction rate can be ≤215.06 mmol m^−2^ s^−1^ at a feed rate of 3 mL min^−1^ and feed concentration of 100 mg L^−1^ [154].

## CHALLENGES AND FUTURE DIRECTIONS

Despite the rapid development of seawater electrocatalysis with ion utilization, several key challenges should be addressed before these methodologies can be deployed at the industrial level. Although current studies have demonstrated proof-of-concept feasibility, the scalability, energy efficiency and long-term stability remain critical bottlenecks.

- Variability of real seawater composition: differing from simulated seawater, natural (real) seawater contains a complex mixture of ions and organic matters. Electrochemical performance is significantly susceptible to seasonal, geographical and environmental fluctuations in the ion concentration of seawater. To address this, pretreatments such as algae removal, sand/turbidity filtration, ultrafiltration and sealing are typically adopted to stabilize the feedwater quality, but they add to the cost input. As many reported studies have shown promising results in artificial seawater but rapidly deteriorate in real seawater, and the pretreatment cost in the overall system is often ignored in real seawater, a more robust and dedicated system design (pretreatment steps, adaptive operation modes, hybrid separation–catalysis schemes and system-level cost analyses) are urgent for real-world operations, even though leveraging the existing seawater desalination infrastructures may offer cost-sharing opportunities.
- The gap between real seawater and simulated seawater: most current studies are conducted in simulated seawater (typically an electrolyte containing only NaCl), which substantially underestimates or neglects the effects of other ions present during seawater electrolysis. As a result, performance data obtained in simulated seawater often fail to represent the true electrochemical behavior in real seawater. Even when real seawater is employed, an alkali (KOH) is added to eliminate the influence of Mg^2+^/Ca^2+^ precipitation at the cathode. In such cases, the research concern is mainly centred on Cl^–^-induced corrosion at the anode, while the corrosion effects arising from other ions remain largely overlooked. Therefore, understanding the effects from other ions during seawater electrolysis is an emerging and urgent direction of both scientific and industrial significance.
- Scalability of chloride-involved reactions: chloride-involved reactions (e.g. hydrocarbon functional and epoxidation) have been demonstrating promising FE values at industrial current densities on a laboratory scale but catalyst deactivation, side reactions and product separation limit their further scaling. Besides, current catalysts are primarily based on noble metals and the design of low-cost catalysts with chlorine resistance and high selectivity is still in the early stages.
- Energy cost of uranium and trace-ion extraction: electrochemical extraction of trace ions (often ppb–ppm levels) is conceptually attractive but remains energy-intensive relative to their market value. For example, although electrochemical uranium extraction has evidenced appreciable selectivity and long-term stability, its current energy demand is underscored, as it is much higher than that of conventional mining. Future research should prioritize improving economic competitiveness: advancing membranes and catalysts with high selectivity and activity to minimize energy waste, coupling with the co-extraction of other ions from seawater to share the energy costs and integrating with waste heat to lower the energy input.
- System-level integration and energy efficiency: although seawater electrocatalysis is a promising concept of coupling hydrogen production and resource recovery, system-level integration design studies remain limited. Furthermore, the energy input is another overlooked factor in the system design, which intuitively needs a practical assessment of energy efficiency, carbon footprint and system-level studies to afford additional energy input. Moreover, electrolyser engineering (membrane optimization, flow-type cell design and product separation) has not flourished in catalyst development. Therefore, an advanced integrated system should prioritize ions/products with high market value or high demand, minimize the additional energy input by coupling with H_2_ production and benchmark energy input/output ratios against industrial standards. Additionally, future smart system-level integration designs should focus on modular and corrosion-resistant architectures that can accommodate different extraction and electrochemical functions, thereby reducing overall capital and operating costs.

## CONCLUSION AND OUTLOOK

Electrocatalysis using water as a proton and hydroxide source faces significant challenges from the inherent presence of additional ions, which can adversely affect electrochemical performance. Historically, research has predominantly focused on mitigating these negative impacts by repelling unwanted ions. However, a crucial paradigm shift is now emerging, emphasizing the strategic utilization of various seawater ions to enhance both the activity and the stability of electrocatalytic processes.

Significant progress has been made in ion utilization within seawater electrocatalysis, broadly categorized into four main strategies: (i) chloride utilization, leveraging chloride through specific adsorption, participation in reactions and immobilization within catalysts to achieve diverse electrochemical outcomes; (ii) sodium utilization, exploiting the conduction effect of sodium, special adsorption properties and hydration mechanisms to optimize reactions; (iii) mechanistic understanding of electrolyser design for magnesium hydroxide co-production focuses on controlling the local pH and introducing external forces on catalysts to facilitate the simultaneous generation of hydrogen and valuable magnesium hydroxide; (iv) electrochemical extraction of valuable ions encompassing the efficient recovery of essential elements such as uranium and lithium from seawater. Despite these advancements, several critical challenges remain, which necessitate a focus of future research and development on the following key areas:

- Chloride utilization: while chloride has shown promise as both a promoter and a protective agent, a limited number of electrocatalysts effectively harness natural seawater chloride. Future research must prioritize the development of materials with intrinsic resistance to chloride attack, whether through direct or indirect utilization. Furthermore, real-time monitoring of chloride utilization remains elusive with the current characterization techniques (e.g. X-ray diffraction (XRD), scanning electron microscope (SEM), XAS), which are primarily result-oriented. The development of advanced *in situ* characterization technologies is paramount to elucidating specific adsorption mechanisms and optimizing chloride-involved reactions. Real-time monitoring of chloride utilization remains a challenge, highlighting the need for advanced *in situ* characterization technologies. This will be a critical area for future research.
- Sodium utilization: the accumulation of sodium during alkaline seawater electrocatalysis positively influences various reactions, such as modulating water configuration in HERs and promoting C–C coupling in CRRs. However, the potential for utilizing this naturally abundant alkaline metal in concentrated seawater for optimizing electrochemical performance remains largely underexplored. Further development of seawater-based cascade electrocatalysis holds immense promise for the sustainable co-production of hydrogen and other value-added chemicals.
- Mechanistic understanding and electrolyser design for hydrogen and magnesium hydroxide co-production: magnesium hydroxide is a valuable co-product of seawater electrocatalysis. However, its co-formation with calcium hydroxide complicates recovery and the precise mechanism for selective magnesium hydroxide generation requires deeper investigation. The mechanism of magnesium hydroxide generation without calcium hydroxide formation is poorly understood and warrants further investigation. Additionally, to enable the efficient recovery of magnesium hydroxide, periodic start–stop implementations are applied to avoid electrolyser blockage. The development of electrolysers specifically designed to produce hydrogen and recover magnesium hydroxide effectively will be essential for practical applications.
- Expanding electrochemical extraction to other valuable seawater ions: seawater contains numerous other valuable ions, including potassium, bromide, sulfate and rubidium, which have widespread applications. For example, potassium can be recovered as potash fertilizers, contributing directly to food security, while bromide can be electrooxidized into valuable bromine or bromine-derived intermediates that are widely applied in fine chemicals and pharmaceutical industries. Although traditional adsorption methods exist for their extraction, electrochemical approaches are significantly underexplored. Developing innovative electrochemical strategies for extracting these ions represents a promising avenue for research, offering potential advancements in sustainability and resource efficiency.

## References

[1] Dinh C-T, Burdyny T, Kibria MG et al. CO_2_ electroreduction to ethylene via hydroxide-mediated copper catalysis at an abrupt interface. Science 2018; 360: 783–7. 10.1126/science.aas9100 29773749

[2] Zhang B, Wang J, Liu G et al. A strongly coupled Ru–CrO*_x_* cluster–cluster heterostructure for efficient alkaline hydrogen electrocatalysis. Nat Catal 2024; 7: 441–51. 10.1038/s41929-024-01126-3

[3] Jiang Y, Shan J, Wang P et al. Stabilizing oxidation state of SnO_2_ for highly selective CO_2_ electroreduction to formate at large current densities. ACS Catal 2023; 13: 3101–8. 10.1021/acscatal.3c00123

[4] Tong W, Forster M, Dionigi F et al. Electrolysis of low-grade and saline surface water. Nat Energy 2020; 5: 367–77. 10.1038/s41560-020-0550-8

[5] Dresp S, Dionigi F, Klingenhof M et al. Direct electrolytic splitting of seawater: opportunities and challenges. ACS Energy Lett 2019; 4: 933–42. 10.1021/acsenergylett.9b00220

[6] Hu H, Zhang Z, Liu L et al. Efficient and durable seawater electrolysis with a V_2_O_3_-protected catalyst. Sci Adv 2024; 10: eadn7012. 10.1126/sciadv.adn7012 38758788 PMC11100561

[7] Fan R, Liu C, Li Z et al. Ultrastable electrocatalytic seawater splitting at ampere-level current density. Nat Sustain 2024; 7: 158–67. 10.1038/s41893-023-01263-w

[8] Duan X, Sha Q, Li P et al. Dynamic chloride ion adsorption on single iridium atom boosts seawater oxidation catalysis. Nat Commun 2024; 15: 1973. 10.1038/s41467-024-46140-y 38438342 PMC10912682

[9] Dionigi F, Reier T, Pawolek Z et al. Design criteria, operating conditions, and nickel-iron hydroxide catalyst materials for selective seawater electrolysis. ChemSusChem 2016; 9:962–72. 10.1002/cssc.201501581 27010750

[10] Zhang S, Wang Y, Li S et al. Concerning the stability of seawater electrolysis: a corrosion mechanism study of halide on Ni-based anode. Nat Commun 2023; 14: 4822. 10.1038/s41467-023-40563-9 37563114 PMC10415325

[11] Bao D, Huang L, Gao Y et al. Dynamic creation of a local acid-like environment for hydrogen evolution reaction in natural seawater. J Am Chem Soc 2024; 146: 34711–9. 10.1021/jacs.4c13036 39573825

[12] Liu Y, Wang Y, Fornasiero P et al. Long-term durability of seawater electrolysis for hydrogen: from catalysts to systems. Angew Chem 2024; 136: e202412087. 10.1002/ange.202412087 39205621

[13] Jin H, Wang X, Tang C et al. Stable and highly efficient hydrogen evolution from seawater enabled by an unsaturated nickel surface nitride. Adv Mater 2021; 33: 2007508. 10.1002/adma.202007508 33624901

[14] Yi L, Chen X, Wen Y et al. Solidophobic surface for electrochemical extraction of high-valued Mg(OH)_2_ coupled with H_2_ production from seawater. Nano Lett 2024; 24: 5920–8. 10.1021/acs.nanolett.4c01484 38708934

[15] Zhao S, Li H, Dai J et al. Selective electrosynthesis of chlorine disinfectants from seawater. Nat Sustain 2024; 7: 148–57. 10.1038/s41893-023-01265-8

[16] Liao H, Luo T, Tan P et al. Unveiling role of sulfate ion in nickel-iron (oxy)hydroxide with enhanced oxygen-evolving performance. Adv Funct Mater 2021; 31: 2102772. 10.1002/adfm.202102772

[17] Ma T, Xu W, Li B et al. The critical role of additive sulfate for stable alkaline seawater oxidation on nickel-based electrodes. Angew Chem Int Ed 2021; 60: 22740–4. 10.1002/anie.202110355 34431193

[18] Li Z, Yao Y, Sun S et al. Carbon oxyanion self-transformation on NiFe oxalates enables long-term ampere-level current density seawater oxidation. Angew Chem Int Ed 2024; 63: e202316522. 10.1002/anie.202316522 37994225

[19] Liu W, Yu J, Sendeku MG et al. Ferricyanide armed anodes enable stable water oxidation in saturated saline water at 2 A/cm^2^. Angew Chem Int Ed 2023; 62: e202309882. 10.1002/anie.202309882 37603411

[20] Kang X, Yang F, Zhang Z et al. A corrosion-resistant RuMoNi catalyst for efficient and long-lasting seawater oxidation and anion exchange membrane electrolyzer. Nat Commun 2023; 14: 3607. 10.1038/s41467-023-39386-5 37330593 PMC10276855

[21] Zhang B, Wang J, Wu B et al. Unmasking chloride attack on the passive film of metals. Nat Commun 2018; 9: 2559. 10.1038/s41467-018-04942-x 29967353 PMC6028649

[22] Kuang Y, Kenney MJ, Meng Y et al. Solar-driven, highly sustained splitting of seawater into hydrogen and oxygen fuels. Proc Natl Acad Sci USA 2019; 116: 6624–9. 10.1073/pnas.1900556116 30886092 PMC6452679

[23] Vos JG, Wezendonk TA, Jeremiasse AW et al. MnO_x_/IrO_x_ as selective oxygen evolution electrocatalyst in acidic chloride solution. J Am Chem Soc 2018; 140: 10270–81. 10.1021/jacs.8b05382 30024752 PMC6099550

[24] Yu L, Zhu Q, Song S et al. Non-noble metal-nitride based electrocatalysts for high-performance alkaline seawater electrolysis. Nat Commun 2019; 10: 5106. 10.1038/s41467-019-13092-7 31704926 PMC6841982

[25] Deng PJ, Liu Y, Liu H et al. Layered double hydroxides with carbonate intercalation as ultra-stable anodes for seawater splitting at ampere-level current density. Adv Energy Mater 2024; 14: 2400053. 10.1002/aenm.202400053

[26] Jin H, Chen J, Qi M et al. Chloride residues in RuO_2_ catalysts enhance its stability and efficiency for acidic oxygen evolution reaction. Angew Chem Int Ed 2025; 64: e202420860. 10.1002/anie.202420860 39794297

[27] Yu Y, Zhou W, Zhou X et al. The corrosive Cl^–^-induced rapid surface reconstruction of amorphous NiFeCoP enables efficient seawater splitting. ACS Catal 2024; 14: 18322–32. 10.1021/acscatal.4c05704

[28] Liu H, Shen W, Jin H et al. High-performance alkaline seawater electrolysis with anomalous chloride promoted oxygen evolution reaction. Angew Chem Int Ed 2023; 62: e202311674. 10.1002/anie.202311674 37711095

[29] Zhuang L, Li J, Wang K et al. Structural buffer engineering on metal oxide for long-term stable seawater splitting. Adv Funct Mater 2022; 32: 2201127. 10.1002/adfm.202201127

[30] Xu W, Wang Z, Liu P et al. Ag nanoparticle-induced surface chloride immobilization strategy enables stable seawater electrolysis. Adv Mater 2024; 36: 2306062. 10.1002/adma.202306062 37907201

[31] Wang Y, Liu Y, Wiley D et al. Recent advances in electrocatalytic chloride oxidation for chlorine gas production. J Mater Chem A 2021; 9: 18974–93. 10.1039/D1TA02745J

[32] Wang Y, Xue Y, Zhang C. Rational surface and interfacial engineering of IrO_2_/TiO_2_ nanosheet arrays toward high-performance chlorine evolution electrocatalysis and practical environmental remediation. Small 2021; 17: 2006587. 10.1002/smll.202006587 33719156

[33] Moreno-Hernandez IA, Brunschwig BS, Lewis NS. Crystalline nickel, cobalt, and manganese antimonates as electrocatalysts for the chlorine evolution reaction. Energy Environ Sci 2019; 12: 1241–8. 10.1039/C8EE03676D

[34] Yang J, Li W-H, Tang H-T et al. CO_2_-mediated organocatalytic chlorine evolution under industrial conditions. Nature 2023; 617: 519–23. 10.1038/s41586-023-05886-z 37198309

[35] Zhang F, Yu L, Wu L et al. Rational design of oxygen evolution reaction catalysts for seawater electrolysis. Trends Chem 2021; 3: 485–98. 10.1016/j.trechm.2021.03.003

[36] Dorakhan R, Grigioni I, Lee B-H et al. A silver–copper oxide catalyst for acetate electrosynthesis from carbon monoxide. Nat Synth 2023; 2: 448–57. 10.1038/s44160-023-00259-w

[37] Lim T, Jung GY, Kim JH et al. Atomically dispersed Pt–N_4_ sites as efficient and selective electrocatalysts for the chlorine evolution reaction. Nat Commun 2020; 11: 412. 10.1038/s41467-019-14272-1 31964881 PMC6972710

[38] Liu Y, Li C, Tan C et al. Electrosynthesis of chlorine from seawater-like solution through single-atom catalysts. Nat Commun 2023; 14: 2475. 10.1038/s41467-023-38129-w 37120624 PMC10148798

[39] Wang S, Xu H, Yao P et al. Ti/RuO_2_-IrO_2_-SnO_2_-Sb_2_O_5_ anodes for Cl_2_ evolution from seawater. Electrochemistry 2012; 80: 507–11. 10.5796/electrochemistry.80.507

[40] Kishor K, Saha S, Parashtekar A et al. Increasing chlorine selectivity through weakening of oxygen adsorbates at surface in Cu doped RuO_2_ during seawater electrolysis. J Electrochem Soc 2018; 165: J3276–80. 10.1149/2.0361815jes

[41] Zhang D, Gong H, Liu T et al. Engineering antibonding orbital occupancy of NiMoO_4_-supported Ru nanoparticles for enhanced chlorine evolution reaction. J Colloid Interface Sci 2024; 672: 423–30. 10.1016/j.jcis.2024.06.023 38850867

[42] Kang C, Li Y, Xu Y et al. Coupling CO_2_-to-ethylene reduction with the chlor-alkaline process in seawater through in situ-formed Cu catalysts. J Phys Chem Lett 2023; 14: 2983–9. 10.1021/acs.jpclett.3c00179 36940469

[43] Zhang X, Wu D, Liu X et al. Efficient electrocatalytic chlorine evolution under neutral seawater conditions enabled by highly dispersed Co_3_O_4_ catalysts on porous carbon. Appl Catal, B 2023; 330: 122594. 10.1016/j.apcatb.2023.122594

[44] Xiao M, Wu Q, Ku R et al. Self-adaptive amorphous CoO*_x_*Cl*_y_* electrocatalyst for sustainable chlorine evolution in acidic brine. Nat Commun 2023; 14: 5356. 10.1038/s41467-023-41070-7 37660140 PMC10475099

[45] Prajapati A, Sartape R, Kani NC et al. Chloride-promoted high-rate ambient electrooxidation of methane to methanol on patterned Cu–Ti bimetallic oxides. ACS Catal 2022; 12: 14321–9. 10.1021/acscatal.2c03619

[46] Leow WR, Lum Y, Ozden A et al. Chloride-mediated selective electrosynthesis of ethylene and propylene oxides at high current density. Science 2020; 368: 1228–33. 10.1126/science.aaz8459 32527828

[47] Lum Y, Huang JE, Wang Z et al. Tuning OH binding energy enables selective electrochemical oxidation of ethylene to ethylene glycol. Nat Catal 2020; 3: 14–22. 10.1038/s41929-019-0386-4

[48] Li A-Z, Yuan B-J, Xu M et al. One-step electrochemical ethylene-to-ethylene glycol conversion over a multitasking molecular catalyst. J Am Chem Soc 2024; 146: 5622–33. 10.1021/jacs.3c14381 38373280

[49] Chung M, Maalouf JH, Adams JS et al. Direct propylene epoxidation via water activation over Pd-Pt electrocatalysts. Science 2024; 383: 49–55. 10.1126/science.adh4355 38175873

[50] Ke J, Zhao J, Chi M et al. Facet-dependent electrooxidation of propylene into propylene oxide over Ag_3_PO_4_ crystals. Nat Commun 2022; 13: 932. 10.1038/s41467-022-28516-0 35177597 PMC8854733

[51] Sun F, Qin J, Wang Z et al. Energy-saving hydrogen production by chlorine-free hybrid seawater splitting coupling hydrazine degradation. Nat Commun 2021; 12: 4182. 10.1038/s41467-021-24529-3 34234135 PMC8263752

[52] Han G, Li G, Sun Y. Electrocatalytic dual hydrogenation of organic substrates with a faradaic efficiency approaching 200%. Nat Catal 2023; 6: 224–33. 10.1038/s41929-023-00923-6

[53] Zhang Q, Chen Y, Yan S et al. Coupling of electrocatalytic CO_2_ reduction and CH_4_ oxidation for efficient methyl formate electrosynthesis. Energy Environ Sci 2024; 17: 2309–14. 10.1039/D4EE00087K

[54] Wang H, Wang S, Song Y et al. Boosting electrocatalytic ethylene epoxidation by single atom modulation. Angew Chem Int Ed 2024; 63: e202402950. 10.1002/anie.202402950 38512110

[55] Wang Q, Li T, Yang C et al. Electrocatalytic methane oxidation greatly promoted by chlorine intermediates. Angew Chem Int Ed 2021; 60: 17398–403. 10.1002/anie.202105523 34060206

[56] Huang L, Wang P, Jiang Y et al. Ethylene electrooxidation to 2-chloroethanol in acidic seawater with natural chloride participation. J Am Chem Soc 2023; 145: 15565–71. 10.1021/jacs.3c05114 37395649

[57] Chung M, Jin K, Zeng JS et al. Mechanism of chlorine-mediated electrochemical ethylene oxidation in saline water. ACS Catal 2020; 10: 14015–23. 10.1021/acscatal.0c02810

[58] Huang L, Bao D, Zheng Y et al. Electrocatalytic production of ethylene glycol from ethylene on both electrodes. ACS Energy Lett 2025; 10: 3907–13. 10.1021/acsenergylett.5c01654

[59] Cai L, Liu Y, Gao Y et al. Atomically asymmetrical Ir–O–Co sites enable efficient chloride-mediated ethylene electrooxidation in neutral seawater. Angew Chem Int Ed 2025; 64: e202417092. 10.1002/anie.202417092 39449650

[60] Li Y, Ozden A, Leow WR et al. Redox-mediated electrosynthesis of ethylene oxide from CO_2_ and water. Nat Catal 2022; 5: 185–92. 10.1038/s41929-022-00749-8

[61] Xue W, Quan L, Liu H et al. Bromine-enhanced generation and epoxidation of ethylene in tandem CO_2_ electrolysis towards ethylene oxide. Angew Chem Int Ed 2023; 62: e202311570. 10.1002/anie.202311570 37699856

[62] Chi M, Ke J, Liu Y et al. Spatial decoupling of bromide-mediated process boosts propylene oxide electrosynthesis. Nat Commun 2024; 15: 3646. 10.1038/s41467-024-48070-1 38684683 PMC11059342

[63] Wang Q, Yang C, Yan Y et al. Electrocatalytic CO_2_ upgrading to triethanolamine by bromine-assisted C_2_H_4_ oxidation. Angew Chem Int Ed 2023; 62: e202212733. 10.1002/anie.202212733 36286347

[64] Quan F, Zhong D, Song H et al. A highly efficient zinc catalyst for selective electroreduction of carbon dioxide in aqueous NaCl solution. J Mater Chem A 2015; 3: 16409–13. 10.1039/C5TA04102C

[65] Zhao M, Tang H, Yang Q et al. Inhibiting hydrogen evolution using a chloride adlayer for efficient electrochemical CO_2_ reduction on Zn electrodes. ACS Appl Mater Interfaces 2020; 12: 4565–71. 10.1021/acsami.9b22811 31909590

[66] Hong S, Lee S, Kim S et al. Anion dependent CO/H_2_ production ratio from CO_2_ reduction on Au electro-catalyst. Catal Today 2017; 295: 82–8. 10.1016/j.cattod.2017.05.063

[67] Varela AS, Ju W, Reier T et al. Tuning the catalytic activity and selectivity of Cu for CO_2_ electroreduction in the presence of halides. ACS Catal 2016; 6: 2136–44. 10.1021/acscatal.5b02550

[68] Gao D, Scholten F, Roldan Cuenya B. Improved CO_2_ electroreduction performance on plasma-activated Cu catalysts via electrolyte design: halide effect. ACS Catal 2017; 7: 5112–20. 10.1021/acscatal.7b01416

[69] Yang P-P, Zhang X-L, Liu P et al. Highly enhanced chloride adsorption mediates efficient neutral CO_2_ electroreduction over a dual-phase copper catalyst. J Am Chem Soc 2023; 145: 8714–25. 10.1021/jacs.3c02130 37021910

[70] Bai S, Song M, Ma T et al. On factors of ions in seawater for CO_2_ reduction. Appl Catal, B 2023; 323: 122166. 10.1016/j.apcatb.2022.122166

[71] Lee S, Kim D, Lee J. Electrocatalytic production of C3-C4 compounds by conversion of CO_2_ on a chloride-induced Bi-phasic Cu_2_O-Cu catalyst. Angew Chem Int Ed 2015; 54: 14701–5. 10.1002/anie.201505730 26473324

[72] Wang H, Matios E, Wang C et al. Rapid and scalable synthesis of cuprous halide-derived copper nano-architectures for selective electrochemical reduction of carbon dioxide. Nano Lett 2019; 19: 3925–32. 10.1021/acs.nanolett.9b01197 31034237

[73] Gao D, Sinev I, Scholten F et al. Selective CO_2_ electroreduction to ethylene and multicarbon alcohols via electrolyte-driven nanostructuring. Angew Chem Int Ed 2019; 131: 17203–9. 10.1002/ange.201910155 PMC6899694 31476272

[74] Kim T, Palmore GTR. A scalable method for preparing Cu electrocatalysts that convert CO_2_ into C_2+_ products. Nat Commun 2020; 11: 3622. 10.1038/s41467-020-16998-9 32681030 PMC7368024

[75] Li M, Ma Y, Chen J et al. Residual chlorine induced cationic active species on a porous copper electrocatalyst for highly stable electrochemical CO_2_ reduction to C_2+_. Angew Chem Int Ed 2021; 60: 11487–93. 10.1002/anie.202102606 33683786

[76] Lu YF, Dong LZ, Liu J et al. Predesign of catalytically active sites via stable coordination cluster model system for electroreduction of CO_2_ to ethylene. Angew Chem Int Ed 2021; 60: 26210–7. 10.1002/anie.202111265 34590413

[77] Dong X, Li S, Zhu C et al. Highly efficient ampere-level CO_2_ reduction to multicarbon products via stepwise hollow-fiber penetration electrodes. Appl Catal, B 2023; 336: 122929. 10.1016/j.apcatb.2023.122929

[78] Hsieh Y-C, Senanayake SD, Zhang Y et al. Effect of chloride anions on the synthesis and enhanced catalytic activity of silver nanocoral electrodes for CO_2_ electroreduction. ACS Catal 2015; 5: 5349–56. 10.1021/acscatal.5b01235

[79] Wan XK, Wang JQ, Wang QM. Ligand-protected Au_55_ with a novel structure and remarkable CO_2_ electroreduction performance. Angew Chem Int Ed 2021; 60: 20748–53. 10.1002/anie.202108207 34288322

[80] Li S, Dong X, Zhao Y et al. Chloride ion adsorption enables ampere-level CO_2_ electroreduction over silver hollow fiber. Angew Chem Int Ed 2022; 61: e202210432. 10.1002/anie.202210432 36056915

[81] Fu HQ, Zhang L, Zheng LR et al. Enhanced CO_2_ electroreduction performance over Cl-modified metal catalysts. J Mater Chem A 2019; 7: 12420–5. 10.1039/C9TA02223F

[82] Hsieh Y-C, Betancourt LE, Senanayake SD et al. Modification of CO_2_ reduction activity of nanostructured silver electrocatalysts by surface halide anions. ACS Appl Energy Mater 2019; 2: 102–9. 10.1021/acsaem.8b01692

[83] Yao J, Yang R, Liu C et al. Alkynes electrooxidation to *α,α*-dichloroketones in seawater with natural chlorine participation via competitive reaction inhibition and tip-enhanced reagent concentration. ACS Cent Sci 2024; 10: 155–62. 10.1021/acscentsci.3c01277 38292614 PMC10823507

[84] Wu B, Lu R, Wu C et al. Pt/IrO*_x_* enables selective electrochemical C-H chlorination at high current. Nat Commun 2025; 16: 166. 10.1038/s41467-024-55283-x 39746984 PMC11696171

[85] Lucky C, Wang T, Schreier M. Electrochemical ethylene oxide synthesis from ethanol. ACS Energy Lett 2022; 7: 1316–21. 10.1021/acsenergylett.2c00265

[86] Wang P, Gao X, Zheng M et al. Urine electrooxidation for energy–saving hydrogen generation. Nat Commun 2025; 16: 2424. 10.1038/s41467-025-57798-3 40069223 PMC11897228

[87] Yan M, Yang R, Liu C et al. *In situ* probing the anion-widened anodic electric double layer for enhanced faradaic efficiency of chlorine-involved reactions. J Am Chem Soc 2025; 147: 6698–706. 10.1021/jacs.4c16173 39953989

[88] Yao J, Cheng C, Wu Y et al. Interfacia hydrogen-bond network regulation tuned water dissociation enables selective chlorination of alkenes. J Am Chem Soc 2025; 147: 8024–31. 10.1021/jacs.5c00818 39976351

[89] Yuan X, Lee K, Schmidt JR et al. Halide adsorption enhances electrochemical hydrogenolysis of 5-hydroxymethylfurfural by suppressing hydrogenation. J Am Chem Soc 2023; 145: 20473–84. 10.1021/jacs.3c06289 37682732

[90] Wang N, Ou P, Hung SF et al. Strong-proton-adsorption co-based electrocatalysts achieve active and stable neutral seawater splitting. Adv Mater 2023; 35: 2210057. 10.1002/adma.202210057 36719140

[91] Sano Y, Hao Y, Kuwahara F. Development of an electrolysis based system to continuously recover magnesium from seawater. Heliyon 2018; 4: e00923. 10.1016/j.heliyon.2018.e00923 30839823 PMC6249789

[92] Shi H, Wang T, Liu J et al. A sodium-ion-conducted asymmetric electrolyzer to lower the operation voltage for direct seawater electrolysis. Nat Commun 2023; 14: 3934. 10.1038/s41467-023-39681-1 37402710 PMC10319863

[93] Ren Y, Fan F, Zhang Y et al. A dual-cation exchange membrane electrolyzer for continuous H_2_ production from seawater. Adv Sci 2024; 11: 2401702. 10.1002/advs.202401702 PMC11220719 38569463

[94] Zhang L, Wang Z, Qiu J. Energy-saving hydrogen production by seawater electrolysis coupling sulfion degradation. Adv Mater 2022; 34: 2109321. 10.1002/adma.202109321 35150022

[95] Liang J, Li J, Dong H et al. Aqueous alternating electrolysis prolongs electrode lifespans under harsh operation conditions. Nat Commun 2024; 15: 6208. 10.1038/s41467-024-50519-2 39043681 PMC11266351

[96] Tan X, Yu C, Song X et al. Toward an understanding of the enhanced CO_2_ electroreduction in NaCl electrolyte over CoPc molecule-implanted graphitic carbon nitride catalyst. Adv Energy Mater 2021; 11: 2100075. 10.1002/aenm.202100075

[97] Wang Y-H, Zheng S, Yang W-M et al. *In situ* raman spectroscopy reveals the structure and dissociation of interfacial water. Nature 2021; 600: 81–5. 10.1038/s41586-021-04068-z 34853456

[98] Bao D, Huang L, Zheng Y et al. Lattice strain-induced regulation of interfacial water promotes hydrogen production from natural seawater. ACS Catal 2025; 15: 14661–70. 10.1021/acscatal.5c03655

[99] Huang L, Bao D, Jiang Y et al. Electrocatalytic acetylene hydrogenation in concentrated seawater at industrial current densities. Angew Chem Int Ed 2024; 63: e202405943. 10.1002/anie.202405943 38769621

[100] Devi N, Wagner A, Lopez J et al. Mechanistic insights into electrodeposition in seawater at variable electrochemical potentials. Adv Sustain Syst 2024; 8: 2300446. 10.1002/adsu.202300446

[101] Carré C, Zanibellato A, Jeannin M et al. Electrochemical calcareous deposition in seawater. a review. Environ Chem Lett 2020; 18: 1193–208.

[102] Liang J, Cai Z, He X et al. Electroreduction of alkaline/natural seawater: self-cleaning Pt/carbon cathode and on-site co-synthesis of H_2_ and Mg hydroxide nanoflakes. Chem 2024; 10: 3067–87. 10.1016/j.chempr.2024.05.018

[103] Liang J, Cai Z, Li Z et al. Efficient bubble/precipitate traffic enables stable seawater reduction electrocatalysis at industrial-level current densities. Nat Commun 2024; 15: 2950. 10.1038/s41467-024-47121-x 38580635 PMC10997793

[104] Kang W, Meng S, Zhao Y et al. Scaling-free cathodes: enabling electrochemical extraction of high-purity nano-CaCO_3_ and -Mg(OH)_2_ in seawater. Environ Sci Technol 2024; 58: 14034–41. 10.1021/acs.est.4c04700 39048519

[105] Zhang X-L, Yu P-C, Sun S-P et al. *In situ* ammonium formation mediates efficient hydrogen production from natural seawater splitting. Nat Commun 2024; 15: 9462. 10.1038/s41467-024-53724-1 39487190 PMC11530463

[106] Wu Y, Xie Y, Liu X et al. Functional nanomaterials for selective uranium recovery from seawater: material design, extraction properties and mechanisms. Coord Chem Rev 2023; 483: 215097. 10.1016/j.ccr.2023.215097

[107] Davies RV, Kennedy J, McIlroy RW et al. Extraction of uranium from sea water. Nature 1964; 203: 1110–5. 10.1038/2031110a0

[108] Tabushi I, Kobuke Y, Nishiya T. Extraction of uranium from seawater by polymer-bound macrocyclic hexaketone. Nature 1979; 280: 665–6. 10.1038/280665a0

[109] Liu C, Hsu P-C, Xie J et al. A half-wave rectified alternating current electrochemical method for uranium extraction from seawater. Nat Energy 2017; 2: 17007. 10.1038/nenergy.2017.7

[110] Tsouris C . Uranium extraction: fuel from seawater. Nat Energy 2017; 2: 17022. 10.1038/nenergy.2017.22

[111] Chi F, Zhang S, Wen J et al. Highly efficient recovery of uranium from seawater using an electrochemical approach. Ind Eng Chem Res 2018; 57: 8078–84. 10.1021/acs.iecr.8b01063

[112] Tang X, Liu Y, Liu M et al. Sulfur edge in molybdenum disulfide nanosheets achieves efficient uranium binding and electrocatalytic extraction in seawater. Nanoscale 2022; 14: 6285–90. 10.1039/D2NR01000C 35411899

[113] Wang C, Helal AS, Wang Z et al. Uranium in situ electrolytic deposition with a reusable functional graphene-foam electrode. Adv Mater 2021; 33: 2102633. 10.1002/adma.202102633 34346102

[114] Jian J, Kang H, Yu D et al. Bi-functional Co/Al modified 1T-MoS_2_/rGO catalyst for enhanced uranium extraction and hydrogen evolution reaction in seawater. Small 2023; 19: 2207378. 10.1002/smll.202207378 36871152

[115] Ismail AF, Yim M-S. Investigation of activated carbon adsorbent electrode for electrosorption-based uranium extraction from seawater. Nucl Eng Technol 2015; 47: 579–87. 10.1016/j.net.2015.02.002

[116] Wang Z, Meng Q, Ma R et al. Constructing an ion pathway for uranium extraction from seawater. Chem 2020; 6: 1683–91. 10.1016/j.chempr.2020.04.012

[117] Wang Z, Ma R, Meng Q et al. Constructing uranyl-specific nanofluidic channels for unipolar ionic transport to realize ultrafast uranium extraction. J Am Chem Soc 2021; 143: 14523–9. 10.1021/jacs.1c02592 34482686

[118] Zhang C, Wang Z, Ma R et al. Overcoming chemical dissociation processes: electrochemical modulation of high-affinity binding sites for rapid uranium extraction from seawater. Adv Funct Mater 2025; 35: 2412712. 10.1002/adfm.202412712

[119] Wang Y, Wang Y, Song M et al. Electrochemical-mediated regenerable Fe^II^ active sites for efficient uranium extraction at ultra-low cell voltage. Angew Chem Int Ed 2023; 62: e202217601. 10.1002/anie.202217601 36905159

[120] Wang W, Xu M, Wu H et al. Precise electrocatalysis on Fe-porphyrin conjugated networks achieves energy-efficient extraction of uranium. Adv Sci 2024; 11: 2409084. 10.1002/advs.202409084 PMC11600223 39373360

[121] Feng H, Dong H, He P et al. Nickel single atom mediated phosphate functionalization of moss derived biochar effectively enhances electrochemical uranium extraction from seawater. J Mater Chem A 2024; 12: 7896–905. 10.1039/D3TA07267C

[122] Chen D, Li Y, Zhao X et al. Self-standing porous aromatic framework electrodes for efficient electrochemical uranium extraction. ACS Cent Sci 2023; 9: 2326–32. 10.1021/acscentsci.3c01291 38161362 PMC10755849

[123] Wang C, Xu M, Wang W et al. A supramolecular organic framework-mediated electrochemical strategy achieves highly selective and continuous uranium extraction. Adv Funct Mater 2024; 34: 2402130. 10.1002/adfm.202402130

[124] Liu X, Xie Y, Hao M et al. Secondary metal ion-induced electrochemical reduction of U(VI) to U(IV) solids. Nat Commun 2024; 15: 7736. 10.1038/s41467-024-52083-1 39231960 PMC11374899

[125] Yang H, Liu X, Hao M et al. Functionalized iron–nitrogen–carbon electrocatalyst provides a reversible electron transfer platform for efficient uranium extraction from seawater. Adv Mater 2021; 33: 2106621. 10.1002/adma.202106621 34599784

[126] Padhi AK, Nanjundaswamy KS, Goodenough JB. Phospho-olivines as positive-electrode materials for rechargeable lithium batteries. J Electrochem Soc 1997; 144: 1188. 10.1149/1.1837571

[127] Zhao Z, Si X, Liu X et al. Li extraction from high Mg/Li ratio brine with LiFePO_4_/FePO_4_ as electrode materials. Hydrometallurgy 2013; 133: 75–83. 10.1016/j.hydromet.2012.11.013

[128] Yang K, Tang M. Three-dimensional phase evolution and stress-induced non-uniform Li intercalation behavior in lithium iron phosphate. J Mater Chem A 2020; 8: 3060–70. 10.1039/C9TA11697D

[129] Yan G, Wang M, Hill GT et al. Defining the challenges of Li extraction with olivine host: the roles of competitor and spectator ions. Proc Natl Acad Sci USA 2022; 119: e2200751119. 10.1073/pnas.2200751119 35878020 PMC9351491

[130] Pasta M, Battistel A, La Mantia F. Batteries for lithium recovery from brines. Energy Environ Sci 2012; 5: 9487. 10.1039/c2ee22977c

[131] Amin R, Balaya P, Maier J. Anisotropy of electronic and ionic transport in LiFePO_4_ single crystals. Electrochem Solid-State Lett 2007; 10: A13. 10.1149/1.2388240

[132] Kim J-S, Lee Y-H, Choi S et al. An electrochemical cell for selective lithium capture from seawater. Environ Sci Technol 2015; 49: 9415–22. 10.1021/acs.est.5b00032 25920476

[133] Sun S, Yu X, Li M et al. Green recovery of lithium from geothermal water based on a novel lithium iron phosphate electrochemical technique. J Cleaner Prod 2020; 247: 119178. 10.1016/j.jclepro.2019.119178

[134] Liu C, Li Y, Lin D et al. Lithium extraction from seawater through pulsed electrochemical intercalation. Joule 2020; 4: 1459–69. 10.1016/j.joule.2020.05.017

[135] Wu L, Zhang C, Kim S et al. Lithium recovery using electrochemical technologies: advances and challenges. Water Res 2022; 221: 118822. 10.1016/j.watres.2022.118822 35834973

[136] Marchini F, Rubi D, Del Pozo M et al. Surface chemistry and lithium-ion xchange in LiMn_2_O_4_ for the electrochemical selective extraction of LiCl from natural salt lake brines. J Phys Chem C 2016; 120: 15875–83. 10.1021/acs.jpcc.5b11722

[137] Trócoli R, Erinmwingbovo C, La Mantia F. Optimized lithium recovery from brines by using an electrochemical ion-pumping process based on λ-MnO_2_ and nickel hexacyanoferrate. ChemElectroChem 2017; 4: 143–9.

[138] Xu X, Zhou Y, Feng Z et al. A self-supported λ-MnO_2_ film electrode used for electrochemical lithium recovery from brines. ChemPlusChem 2018; 83: 521–8. 10.1002/cplu.201800185 31950655

[139] Xie N, Li Y, Lu Y et al. Electrochemically controlled reversible lithium capture and release enabled by LiMn_2_O_4_ nanorods. ChemElectroChem 2020; 7: 105–11. 10.1002/celc.201901728

[140] Lawagon CP, Nisola GM, Cuevas RAI et al. Li_1−_*_x_*Ni_0.33_Co_1/3_Mn_1/3_O_2_/Ag for electrochemical lithium recovery from brine. Chem Eng J 2018; 348: 1000–11. 10.1016/j.cej.2018.05.030

[141] Wu Y, Shi P, Zhong Y et al. Improved performance of a Ni, Co-Doped LiMn_2_O_4_ electrode for lithium extraction from brine. Energy Fuels 2023; 37: 4083–93. 10.1021/acs.energyfuels.2c04113

[142] Guan D, Jeevarajan JA, Wang Y. Enhanced cycleability of LiMn_2_O_4_ cathodes by atomic layer deposition of nanosized-thin Al_2_O_3_ coatings. Nanoscale 2011; 3: 1465. 10.1039/c0nr00939c 21327283

[143] Luo G, Zhu L, Li X et al. Electrochemical lithium ions pump for lithium recovery from brine by using a surface stability Al_2_O_3_–ZrO_2_ coated LiMn_2_O_4_ electrode. J Energy Chem 2022; 69: 244–52. 10.1016/j.jechem.2022.01.012

[144] Zhao X, Jiao Y, Xue P et al. Efficient lithium extraction from brine using a three-dimensional nanostructured hybrid inorganic-gel framework electrode. ACS Sustain Chem Eng 2020; 8: 4827–37. 10.1021/acssuschemeng.9b07644

[145] Shang X, Liu J, Hu B et al. CNT-strung LiMn_2_O_4_ for lithium extraction with high selectivity and stability. Small Methods 2022; 6: 2200508. 10.1002/smtd.202200508 35560872

[146] Suss ME, Baumann TF, Bourcier WL et al. Capacitive desalination with flow-through electrodes. Energy Environ Sci 2012; 5: 9511. 10.1039/c2ee21498a

[147] Oren Y . Capacitive deionization (CDI) for desalination and water treatment—past, present and future (a review). Desalination 2008; 228: 10–29. 10.1016/j.desal.2007.08.005

[148] Bouhadana Y, Avraham E, Soffer A et al. Several basic and practical aspects related to electrochemical deionization of water. AlChE J 2010; 56: 779–89. 10.1002/aic.12005

[149] Johnson AM, Newman J. Desalting by means of porous carbon electrodes. J Electrochem Soc 1971; 118: 510. 10.1149/1.2408094

[150] Zhang C, He D, Ma J et al. Comparison of faradaic reactions in flow-through and flow-by capacitive deionization (CDI) systems. Electrochim Acta 2019; 299: 727–35. 10.1016/j.electacta.2019.01.058

[151] Johnson A, Venolia A, Newman J et al. Electrosorb process for desalting water, office of saline water research and development progress report no. 516, US Dept. Interior Pub 1970; 200: 31.

[152] Porada S, Shrivastava A, Bukowska P et al. Nickel hexacyanoferrate electrodes for continuous cation intercalation desalination of brackish water. Electrochim Acta 2017; 255: 369–78. 10.1016/j.electacta.2017.09.137

[153] Dahiya S, Mishra BK. Enhancing understandability and performance of flow electrode capacitive deionisation by optimizing configurational and operational parameters: a review on recent progress. Sep Purif Technol 2020; 240: 116660. 10.1016/j.seppur.2020.116660

[154] Ha Y, Jung HB, Lim H et al. Continuous lithium extraction from aqueous solution using flow-electrode capacitive deionization. Energies 2019; 12: 2913. 10.3390/en12152913

